# Functional and phylogenetic responses of motile cryptofauna to habitat degradation

**DOI:** 10.1111/1365-2656.13809

**Published:** 2022-09-11

**Authors:** Jessica S. Stella, Kennedy Wolfe, George Roff, Alice Rogers, Mark Priest, Yimnang Golbuu, Peter J. Mumby

**Affiliations:** ^1^ The Great Barrier Reef Marine Park Authority Townsville Queensland Australia; ^2^ Marine Spatial Ecology Lab, School of Biological Sciences and ARC Centre of Excellence for Coral Reef Studies University of Queensland St Lucia Queensland Australia; ^3^ Victoria University of Wellington, School of Biological Sciences Wellington New Zealand; ^4^ Palau International Coral Reef Center Koror Palau

**Keywords:** complexity, coral, cryptic, diversity, invertebrate, rubble, trophodynamics, water quality

## Abstract

1. Biodiversity of terrestrial and marine ecosystems, including coral reefs, is dominated by small, often cryptic, invertebrate taxa that play important roles in ecosystem structure and functioning. While cryptofauna community structure is determined by strong small‐scale microhabitat associations, the extent to which ecological and environmental factors shape these communities are largely unknown, as is the relative importance of particular microhabitats in supporting reef trophodynamics from the bottom up.
2. The goal of this study was to address these knowledge gaps, provided coral reefs are increasingly exposed to multiple disturbances and environmental gradients that influence habitat complexity, condition and ecosystem functioning.
3. We compared the density, biomass, size range, phylogenetic diversity and functional roles of motile cryptofauna in Palau, Western Micronesia, among four coral‐derived microhabitats representing various states of degradation (live coral [*Acropora* and *Pocillopora*], dead coral and coral rubble) from reefs along a gradient of effluent exposure.
4. In total, 122 families across ten phyla were identified, dominated by the Arthropoda (Crustacea) and Mollusca. Cryptofauna biomass was greatest in live *Pocillopora*, while coral rubble contained the greatest density and diversity. Size ranges were broader in live corals than both dead coral and rubble. From a bottom‐up perspective, effluent exposure had mixed effects on cryptic communities including a decline in total biomass in rubble. From a top‐down perspective, cryptofauna were generally unaffected by predator biomass.
5. Our data show that, as coral reef ecosystems continue to decline in response to more frequent and severe disturbances, habitats other than live coral may become increasingly important in supporting coral reef biodiversity and food webs.

## Graphical abstract

Uncovering the hidden biodiversity of coral reefs. Divers mostly see corals and fishes, but these authors have found increased numbers and diversity of tiny animals hidden in dead coral and rubble. Such animals are overlooked, but may help support food webs and functioning as the living coral structure degrades.

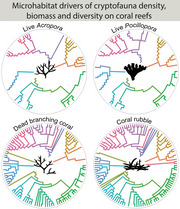

## 1 INTRODUCTION

Animal diversity of Earth's ecosystems is dominated by the Invertebrata (Wilson, 1992), where, despite their small size, they are critical to ecosystem functioning (Hättenschwiler et al., 2005; Lassau et al., 2005; Lawrence & Britton, 1994; Weisser & Siemann, 2004). Invertebrates play a key role in terrestrial food webs across multiple trophic levels (Wilson, 1992), and keystone taxa such as leaf‐cutting ants and mound‐building termites can drive profound changes to both biotic and abiotic processes (Elmes, 1991; Hölldobler & Wilson, 1990; Jones et al., 1994; Lugo et al., 1973). The composition and distribution of invertebrate communities in terrestrial systems, such as woodlands and forests, are partly regulated by habitat complexity (Halaj et al., 2000; Lassau et al., 2005; Wardhaugh et al., 2013), and habitat fragmentation and loss at landscape scales can have pervasive effects on biodiversity, ecosystem functioning and food web structuring (Fahrig, 2003; Leal et al., 2014; Tscharntke et al., 2002; Valladares et al., 2012).

The role of invertebrates in marine ecosystems is not as well characterised as in terrestrial ecosystems. Of the most biodiverse marine ecosystems—coral reefs—the majority of studies have targeted corals and reef fishes (Bellwood et al., 2017; Fisher et al., 2015; Przeslawski et al., 2008; Reaka‐Kudla, 1997). Yet, animal species diversity in coral reefs is dominated by cryptic organisms (the cryptofauna) that take refuge inside the complexities of the reef matrix (Carvalho et al., 2019; Castro, 1988; Glynn & Enochs, 2011; Hutchings, 1983; Peyrot‐Clausade, 1979; Plaisance et al., 2011; Wolfe et al., 2021). Motile cryptofauna, including worms, molluscs and arthropods (crustaceans), are integral components of food webs and function on coral reefs, contributing to filter feeding and water quality (Richter & Wunsch, 1999), detrital recycling (Depczynski & Bellwood, 2003), microherbivory (Altman‐Kurosaki et al., 2018; Brawley & Adey, 1981; Klumpp et al., 1988), and bioerosion (Hutchings, 1986). Reef‐associated invertebrates account for >90% of the known fauna on coral reefs (Przeslawski et al., 2008; Reaka‐Kudla, 1997; Stella et al., 2010), and cryptobenthic fishes represent around half the total number of reef fishes (Brandl et al., 2018; Depczynski & Bellwood, 2003). Cryptofauna are a critical food source across a broad range of trophic levels (Glynn & Enochs, 2011; Opitz, 1993), with cryptobenthic fishes producing almost 60% of consumed reef fish biomass (Brandl et al., 2019; Goatley & Brandl, 2017). Such high productivity highlights the importance of the cryptofauna to reef trophodynamics, providing direct links from the benthos to higher‐order consumers (Depczynski & Bellwood, 2003; Hiatt & Strasburg, 1960; Hutchings, 1983; Rinkevich et al., 1991; Rogers, Blanchard, & Mumby, 2018; Rogers, Blanchard, Newman, et al., 2018).

Density and diversity of cryptofauna are largely dependent on microhabitat structure, which offers refuge from predation and facilitate biological functioning inside the reef framework (Enochs et al., 2011). Live branching corals (e.g. *Acropora* and *Pocillopora*) offer high habitat (reef scale) and microhabitat (colony scale) complexity and thereby support vast, often specialised, assemblages, particularly for the Crustacea (Stella, Pratchett, et al., 2011). Microhabitat characteristics are highly variable across coral reefs, and are largely a physical reflection of the degradation process from live coral to dead coral, to rubble and sand (Hutchings, 1986). Disturbance regimes are therefore an important determinant of the structure of the cryptobiome and marine food webs (Connell, 1978; Enochs et al., 2011; Enochs & Manzello, 2012b; Gonzalez‐Gomez et al., 2018; Head et al., 2018; Klumpp et al., 1988; Kramer et al., 2014b; Milazzo et al., 2019; Moran & Reaka‐Kudla, 1991; Nelson et al., 2016; Takada et al., 2016; Wilson et al., 2006). While high coral cover is often hailed as a hallmark of a healthy reef ecosystem, rising pressures on coral reefs (Wolff et al., 2015) and declining coral populations (Hughes, Anderson, et al., 2018) highlight the need to understand how ‘degraded’ microhabitats (i.e. dead coral and rubble) support ecosystem processes and functions through enhanced biodiversity and resource availability from the bottom‐up (Enochs & Manzello, 2012a; Rogers et al., 2015; Wolfe et al., 2021).

Ecologists have long debated the importance of trophic interactions and the effects of resources (bottom‐up control) and predators (top‐down control) in determining the abundance, distribution and functioning of reef organisms (Hairston et al., 1960; Hunter & Price, 1992; Power, 1992). In coral reef ecosystems, invertivorous fishes (and cryptopredators) represent a critical trophic link in top‐down control, moving energy from the benthos to the water column (Depczynski & Bellwood, 2003; Goatley et al., 2017; Kramer et al., 2015; Rogers, Blanchard, & Mumby, 2018). Invertivores can be the most speciose group on coral reefs (Kramer et al., 2015; Williams & Hatcher, 1983; Wolfe, Anthony, et al., 2020), and many have evolved specialised morphologies related to foraging or dietary niches (Bellwood et al., 2010; Hiatt & Strasburg, 1960; Hobson, 1974). From a bottom‐up perspective, cryptofauna collectively use a broad range of microhabitats and resources (Altman‐Kurosaki et al., 2018; Brandl et al., 2018; Glynn & Enochs, 2011; Goatley et al., 2017; Hutchings, 1983; Wolfe et al., 2021). Bottom‐up changes on coral reefs, such as a decline in water quality or habitat, have the potential to shift trophodynamics, especially since some cryptic populations are dependent on microhabitat availability (Stella, Pratchett, et al., 2011) and on local mobility and self‐seeding rather than external recruitment (Moran & Reaka‐Kudla, 1991; Takada et al., 2016). Yet, the ecosystem‐scale impacts of declining water quality and habitat condition on the cryptobiome remain largely unknown.

Here, we explore the diversity and functional relationships of cryptofauna in a Pacific coral reef system (Palau, Western Micronesia). To date, most studies on cryptofauna in coral reefs have focused on particular microhabitat types (e.g. Counsell et al., 2018; Head et al., 2015; Klumpp et al., 1988) or key taxonomic groups (e.g. Ahmadia et al., 2012; Gonzalez‐Gomez et al., 2018; Head et al., 2018; Kramer et al., 2014a), though eDNA metabarcoding techniques are showing promise partitioning the cryptobiome (Carvalho et al., 2019; Leray & Knowlton, 2015; Pearman et al., 2018; Plaisance et al., 2009). We characterise the density, diversity and biomass of cryptofauna across four common habitat types and explore the relationship between top‐down (i.e. predation by invertivorous reef fishes) and bottom‐up (i.e. water quality, habitat) drivers on the cryptofauna. We explore the implications of habitat degradation (live coral–dead coral–rubble) for reef biodiversity in the face of rapid global change, predicting that cryptobiomes may be transformed by homogenisation of habitat‐associated fauna.

## 2 MATERIALS AND METHODS

### 2.1 Study site

**FIGURE 1:**
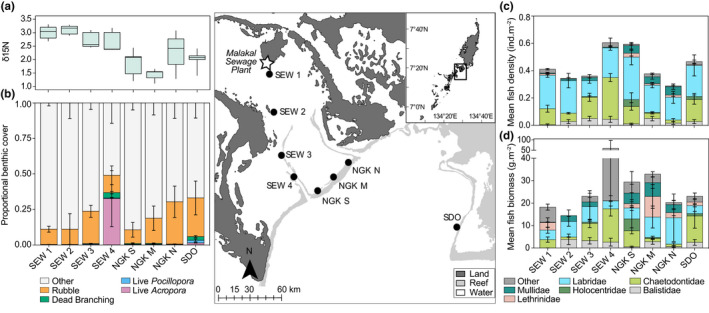
Indication of the eight survey sites (black dots) on the east coast of Palau, including effluent source (Malakal Sewage Plant, white star), and site characterisation including bottom‐up (a) water quality (see Roff et al., 2019) and (b) proportional benthic cover, and top‐down invertivorous reef fish (c) density and (d) biomass drivers at each site.

This study was conducted in October–December 2014 on the east coast of Palau, Western Micronesia (Figure 1). Eight sites were established along a water quality gradient resulting from proximity to the Malakal Sewage Plant outlet (Figure 1). Herein, we refer to the four sites closest to the effluent outlet as SEW 1 (closest) to SEW 4 (farthest), the three sites on Ngderrak Reef as NGK N (north), NGK M (mid) and NGK S (south), and furthest site across the deep‐water channel as SDO (known locally as Short Drop Off) (Figure 1). Nitrogen data (δ15^N^) were taken from Roff et al. (2019) as a proxy for water quality at each site (Figure 1a), as determination of δ15^N^ is a method used to detect nutrient input and sewage pollution (Costanzo et al., 2001). We then characterised the percent cover of coral‐derived microhabitats (Figure 1b; live *Acropora*, live *Pocillopora*, dead coral and coral rubble) and invertivorous fish assemblages (Figure 1c,d) at each site to explore primary drivers of variation in cryptofauna communities.

### 2.2 Benthic composition

Surveys of benthic substrata were conducted at ~7 m depth at each site to determine the proportion of the benthos comprising the four main microhabitats; live branching pocilloporids, live branching acroporids, dead branching coral and coral rubble. These microhabitats are key to the diversity of cryptobenthic communities on coral reefs (Head et al., 2015, 2018; Klumpp et al., 1988; Kramer et al., 2014b; Stella et al., 2010; Stella, Pratchett, et al., 2011). As our focus was on microhabitats derived from branching coral, additional benthic components (e.g. sand, algae, other coral taxa) were collectively classified as ‘other’, but this does not negate their importance in supporting diverse cryptofauna communities (e.g. Chen et al., 2020; Tano et al., 2016). To determine proportional benthic cover of each microhabitat, 24 replicate 1 m^2^ quadrats were randomly placed on the substrate and photographed at each site. Images were later analysed using Vidana (https://www.marinespatialecologylab.org/resources‐1), and the average proportion of each microhabitat was calculated per site.

### 2.3 Invertivorous reef fish assemblages

Density and biomass of invertivorous reef fishes were determined at each site and used here as a proxy for top‐down control on benthic cryptofauna. Direct measures of predation (e.g. gut contents or bite rates) were not acquired. Invertivore density was examined along three replicate belt transects (30 × 2 m) per site over four days. All surveys were conducted in daylight hours. Nocturnally active invertivores were excluded. All individuals known to include invertebrates in their diet were identified to the species level and their size estimated to the nearest centimetre. We determined the efficiency of sampling using the metric of precision (percentage of standard error to mean), which should ideally be <20% (Andrew & Mapstone, 1987). The precision of observed invertivore density per site (i.e. among density estimates from replicate transects) did not exceed 20% at any site (mean site‐level precision 13% ± 1.8% SE). Thus, using transects of 30 × 20 m with a sample size of three was sufficient to obtain precise estimates of invertivore density.

Fish biomass estimates were calculated using species‐specific length‐to‐weight data (Table S1) from FishBase (Froese & Pauly, 2019). Mean density (ind.m^−2^) and biomass (g.m^−2^) of invertivorous coral reef fishes were calculated by family for each site. Refer to Table S1 for a full list of the observed invertivorous coral reef fish species. Ethical approval to survey fish assemblages was provided under project 2014/AE000038 of the University of Queensland Ethical Approval Process. This research was carried out as part of the Palau International Coral Reef Center's research portfolio.

### 2.4 Cryptofauna assemblages

Three replicate samples of each microhabitat were collected from each site between 5–7 m depth. Live (*Acropora*, *Pocillopora*) and dead (*Acropora*) coral colonies were collected from each site by covering the entire colony with a plastic bag to prevent any mobile organisms from escaping, and the entire intact colony was then carefully chiselled free of the substrate (e.g. Klumpp et al., 1988). Colonies of ~20 cm in diameter were targeted as a first quality control for size standardisation of microhabitats. Despite low coral cover at sites dominated by rubble (see Figure 1), live coral was sufficiently abundant to collect three replicates of *Acropora* and *Pocillopora* in almost all cases. Live *Acropora* were absent from one site (NGK S) and thus, were not collected at this site. Live *Acropora* was therefore represented by *n* = 21 samples from seven sites, while the three remaining microhabitats are represented by *n* = 24 samples from eight sites. Coral rubble samples were collected from each site by inserting open plastic mesh trays (22 × 18 × 5.8 cm, mesh size 64 mm^2^) filled with defaunated rubble in place of a natural rubble patch. All rubble pieces were derived from dead branching coral fragments. The rubble containers were left in the field for ~7 days to allow recolonisation, a common approach used to sample rubble‐dwelling fauna (Wolfe et al., 2021). Upon retrieval, trays were lifted from their depression and placed into plastic bags. Once collected, all communities were transferred into individual buckets of fresh seawater and transported back to the laboratory within 2 h for processing following the methods described in Stella et al. (2010).

In brief, conspicuous individuals were partitioned before submerging each replicate substrate in fresh water for <1 min to expel cryptic organisms. The water was poured through a 1 × 1 mm mesh net to retrieve all remaining individuals. This method was effective in capturing macrofauna (>1 mm), but excluded microfauna (<1 mm), meaning the total density and diversity of organisms documented here is underestimated. All individuals were identified to the highest taxonomic resolution possible under a dissecting microscope based on all current literature (e.g. WoRMS: www.marinespecies.org; Zootaxa: www.biotaxa.org/Zootaxa) and with the assistance of taxonomic consultants. All individuals were then measured to the nearest 0.1 mm and weighed to the nearest mg on an analytical balance (AND GR‐120). Standard measurements were used; carapace width for crab‐like crustaceans or length for shrimp‐like crustaceans, shell length for molluscs (longest distance), diameter for echinoderms with radial symmetry, and length for all types of worms. Residual water was blotted off before weight was recorded. Motile cryptofauna density (m^−3^) and biomass (g.m^−3^) were standardised to the volume (as measured by the water displacement method) of their respective microhabitat sample (mean ± SE); live *Acropora* (3235 ± 470 ml^3^), live *Pocillopora* (4405 ± 483 ml^3^), dead branching coral (4037 ± 479 ml^3^) and rubble (1487 ± 42 ml^3^). Average density (ind.m^−3^) and biomass (g.m^−3^) were then quantified by family for each microhabitat‐site combination (*n* = 3).

### 2.5 Functional traits and phylogenies of the cryptofauna

**FIGURE 2:**
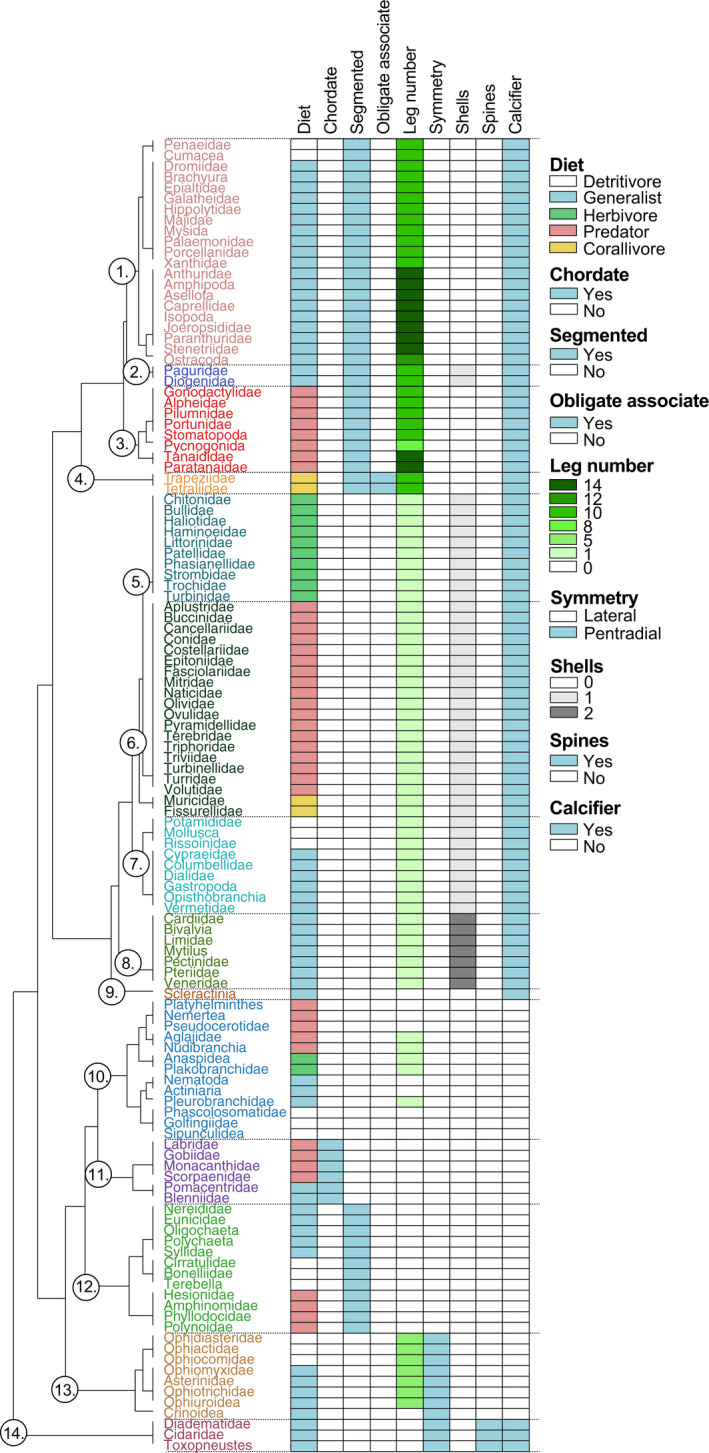
Functional groups resulting from the trait‐based assessment of the 122 families of motile cryptofauna identified on the east coast of Palau: (1) Primary crustaceans, (2) hermit crabs, (3) secondary crustaceans, (4) specialist crustaceans, (5) primary gastropods, (6) secondary gastropods, (7) other gastropods, (8) bivalves, (9) hard coral, (10) soft‐bodied invertebrates, (11) fishes, (12) segmented worms, (13) non‐calcified echinoderms and (14) sea urchins.

Taxonomic information and phylogenetic relationships among each cryptofaunal family were obtained from the Tree of Life database (Maddison & Schulz, 2007). Functional classification of each family was performed using a traits‐based assessment on nine biological and ecological traits: (1) diet (herbivore, detritivore, generalist including filter‐feeders and mixed‐diets, corallivore or predator), (2) chordate (yes, no), (3) segmented (yes, no), (4) habitat association (generalist or obligate), (5) leg number, (6) body plan (symmetry) (yes, no), (7) the presence of spines (yes, no), (8) shell number and (9) calcifier (yes, no); summarised in Figure 2. Traits considered more subjective at the family level (e.g. diet and habitat association) were determined from published literature and online databases (e.g. Tree of Life, as above). Functional traits were clustered using a Gowers dissimilarity measure for mixed variables (Gower, 1971) using the *gowdis* function in fd package in r (Laliberté & Legendre, 2010), and plotted using an average unweighted hierarchical cluster analysis using the *hclust* function in stats package in r (R Core Team, 2019).

### 2.6 Data analysis

Differences in benthic composition (microhabitat cover) and invertivorous fish assemblages among sites were examined using permutational analysis of variance (PERMANOVA) in PRIMER (v6) with 999 permutations using Euclidean distances (Anderson et al., 2008). Site was used as a fixed factor and either proportional benthic composition, invertivore density or invertivore biomass as the response variable. Significant differences were explored further using pairwise tests. Data were log‐transformed before analysis. Data on water quality (δ15^N^) were published (Roff et al., 2019) and were not analysed here.

A phylogenetic tree was constructed at the highest level of taxonomy possible from the open Tree of Life nodes using the *ggtree* function in r (Yu et al., 2017). Shannon‐Weaver Diversity Index, Simpson's Diversity Index (inverse) and Pielou's Evenness Coefficient were calculated for each microhabitat using the *diversity* function of the vegan package in r (Oksanen et al., 2019).

Mixed‐effects PERMANOVAs were used to examine differences in cryptofauna density (ind.m^−3^) and biomass (g.m^−3^) using Bray–Curtis distribution matrices, with site and microhabitat as fixed factors. Water quality, proportional benthic cover and invertivorous fish biomass (g.m^−2^) were considered covariates. Cryptofauna density and biomass were log‐transformed prior to analysis. Contributions to significant differences in cryptofauna density and biomass were explored using pairwise and similarity percentage analysis (SIMPER) tests. Size distributions of the cryptofauna were analysed by microhabitat using the Kruskal–Wallis and pairwise Wilcoxon rank sum tests in the stats package of r (R Core Team, 2019). Community structure in each microhabitat was further examined using principle component analysis (PCA) using the *ade4* and *factoextra* packages in R (Dray & Dufour, 2007; Kassambara & Mundt, 2017). Analyses were performed for cryptofauna by family, then by functional group.

Linear models were used to explore relationships between each functional group and water quality, benthic cover and invertivorous fish biomass. Correlations between these factors and biodiversity coefficients (i.e. Shannon‐Weaver Diversity Index, Simpson's Diversity Index (inverse), Pielou's Evenness Coefficient) were also examined. Linear models were performed for density (log transformed) using the *lm* and *step* functions in the *stats* package of R (Chambers & Hastie, 1992; R Core Team, 2019). Normality and homogeneity of variance were explored using residual and quantile‐quantile plots using the ggfortify package (Tang et al., 2016).

## 3 RESULTS

### 3.1 Benthic composition

Benthic composition differed among the eight sites, which was dominated by the benthic category ‘other’ (Figure 1b; Table S3). Of the four microhabitats examined, coral rubble predominated all sites with the exception of SEW 4, which was dominated by live *Acropora* (Figure 1b; Tables S3). Live coral cover (*Acropora* and *Pocillopora*) was an order of magnitude greater at this site (33 ± 19%, mean ± SE) compared to the site furthest from the sewage outfall (SDO; 3.2 ± 2.0%) and was around two orders of magnitude greater than the remaining sites (0.1–0.6%) (Figure 1b). Live coral cover was generally low (Figure 1b), but regardless of their relative abundance at each site, the four microhabitats considered in this study were sufficiently abundant to sample from all sites, with the exception of NGK S where no live *Acropora* was found or sampled (Figure 1b).

### 3.2 Invertivorous reef fish assemblages

Seventy species from 14 families of invertivorous coral reef fishes were identified (Table S1). Differences were found in the density (Mean Sum of Squares [MS] = 4.34, *p* < 0.001) and biomass (MS = 64.0, *p* = 0.01) of invertivores among the eight study sites (Figure 1c,d, Table S3), with the greatest densities at NGK S and SEW 4, and lowest at NGK N (Figure 1c). Invertivorous reef fish biomass was greatest at SEW 4 (Figure 1d).

### 3.3 Taxonomic composition of the cryptofauna

**FIGURE 3:**
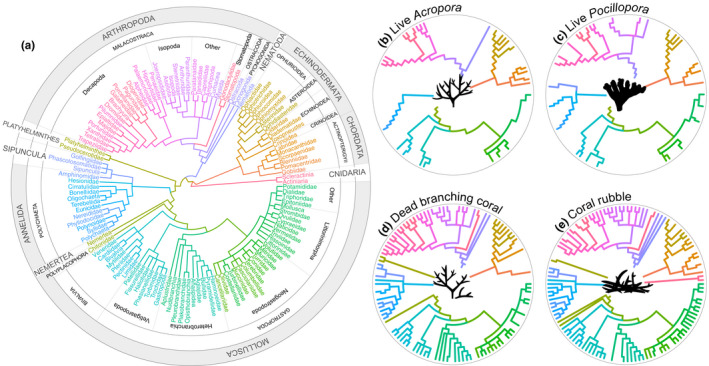
Phylogenetic trees expressing lineages of (a) all motile cryptofauna identified (predominantly to family), including subsets for communities occupying (b) live *Acropora*, (c) live *Pocillopora*, (d) dead branching coral and (e) coral rubble. Main plot: Outer ring = phylum; middle ring = class, inner ring = order/subclass.

At least 122 families were identified across ten invertebrate and vertebrate phyla (Figures 2 and 3; Table S2). Diversity was best represented in the Mollusca (51 of 122 families) and Arthropoda (33 of 122 families) (Figure 3). The proportion of total species diversity was greatest in coral rubble (103 of 122 families), followed by dead coral (91 of 122 families), and was lowest in both live *Acropora* and *Pocillopora* (31 of 122 families) (Figure 3, Table 1). Conversely, species evenness was greatest in live coral (Table 1).

**TABLE 1:** Biodiversity indices for motile cryptofauna occupying microhabitats on the east coast of Palau. Indices include total abundance (*N*), mean abundance with standard error (*XN* ± SE), Shannon‐Weaver diversity index (*H′*), Simpson's diversity index (inverse) (1/*λ*), and Pielou's species evenness coefficient (*J'*)

Habitat	*N*	*XN* ± SE	*H′*	1/*λ*	*J'*
Live *Acropora*	166	7.90 ± 1.33	1.17 ± 0.11	3.23 ± 0.35	0.89 ± 0.02
Live *Pocillopora*	458	19.08 ± 2.18	1.59 ± 0.06	4.14 ± 0.23	0.87 ± 0.01
Dead coral	1669	69.54 ± 8.24	2.35 ± 0.07	7.62 ± 0.64	0.82 ± 0.02
Coral rubble	2202	91.75 ± 5.31	2.85 ± 0.05	12.50 ± 0.70	0.87 ± 0.01

**FIGURE 4:**
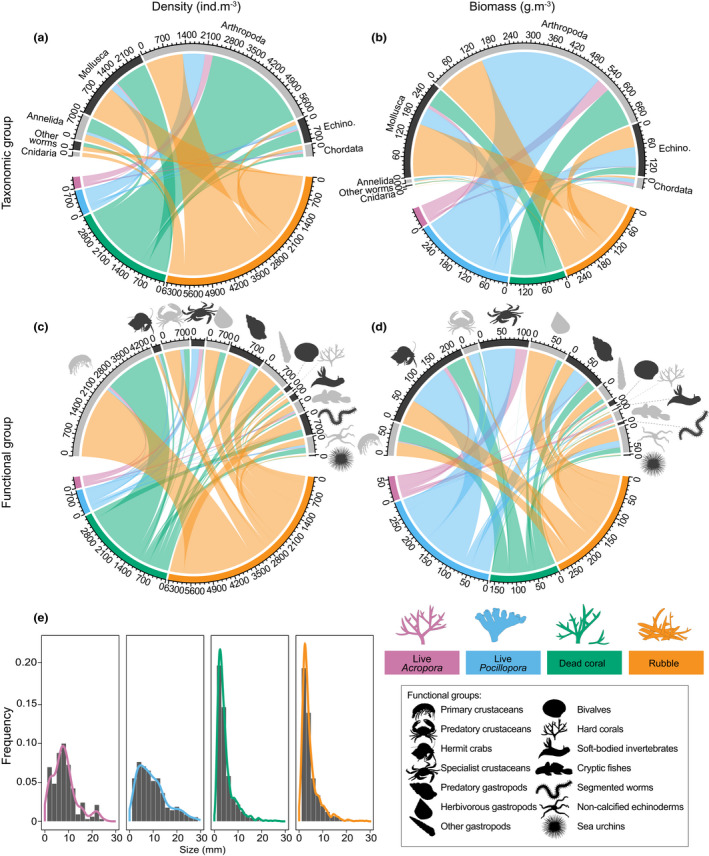
Chord plots depicting average (a, c) density (ind.m^−3^) and (b, d) biomass (g.m^−3^) of motile cryptofauna occupying live *Acropora* (purple), live *Pocillopora* (blue), dead coral (green) and coral rubble (orange) (*n* = 24; except *n* = 21 for live *Acropora*, see Section 2) from taxonomic (a, b) and functional group (c, d) perspectives, as well as (e) cryptofauna size distribution per habitat. For chord plots, colours represent microhabitat, and the arc and node sizes represent relative magnitude from microhabitats (bottom nodes) to cryptofauna (upper nodes).

Arthropoda (Crustacea) and Mollusca dominated samples representing 55% and 28% of the total density and 58% and 30% of the total biomass, respectively (Figure 4a,b). Cryptic community density differed among the four microhabitat types and eight study sites (Figure 4a, Tables 2, Tables S4 and S5). Covariates of water quality and benthic cover significantly influenced the density and biomass of motile cryptofauna at a community level, while invertivore biomass did not (Table 2). Dead coral and rubble hosted an order of magnitude or more cryptic individuals than live *Acropora*, and 4–5 times the densities associated with live *Pocillopora* (Figure 4a, Table 1). Coral rubble hosted 49% of the total number of individuals counted, while intact dead coral hosted 37% of the total count (Figure 4a, Table 1).

**TABLE 2:** PERMANOVA results on the density (m^−3^) and biomass (g.m^−3^) of motile cryptofauna by taxonomy and functional group across survey sites and microhabitat types on the east coast of Palau. Significant values in bold

Source	df	SS	MS	Pseudo‐*F*	*p*‐perm	Unique perms
***Density–Taxonomy***						
*δ15* ^*N*^	1	4854	4854	3.78	**0.001**	997
*Cover*	1	23,466	23,466	18.29	**0.001**	998
*Biomass*	1	1499	1499	1.17	0.316	999
*Site*	5	15,855	3171	2.47	**0.001**	999
*Habitat*	3	108,640	36,212	28.22	**0.001**	999
*Si × Ha*	19	33,891	1784	1.39	**0.002**	998
*Res*	62	79,558	1283			
*Total*	92	267,760				
***Biomass–Taxonomy***						
*δ15* ^*N*^	1	6764	6764	4.61	**0.001**	997
*Cover*	1	19,704	19,704	13.43	**0.001**	998
*Biomass*	1	1821	1821	1.24	0.238	998
*Site*	5	14,076	2815	1.92	**0.001**	996
*Habitat*	3	105,820	35,274	24.04	**0.001**	998
*Si × Ha*	19	34,369	1809	1.23	**0.01**	998
*Res*	62	90,988	1468			
*Total*	92	273,540				
***Density–Functional group***						
*δ15* ^*N*^	1	2193	2193	4.33	**0.003**	998
*Cover*	1	15,483	15,483	30.56	**0.001**	999
*Biomass*	1	19.6	19.6	0.04	0.924	998
*Site*	5	6903	1381	2.73	**0.002**	998
*Habitat*	3	62,297	20,766	40.99	**0.001**	998
*Si × Ha*	19	13,257	698	1.38	0.053	998
*Res*	62	31,407	507			
*Total*	92	131,560				
***Biomass–Functional group***						
*δ15* ^*N*^	1	3834	3834	5.45	**0.001**	998
*Cover*	1	13,303	13,303	18.90	**0.001**	999
*Biomass*	1	84	84	0.12	0.929	998
*Site*	5	8706	1741	2.47	**0.001**	997
*Habitat*	3	73,318	24,439	34.73	**0.001**	999
*Si × Ha*	19	19,645	1034	1.47	**0.014**	999
*Res*	62	43,630	104			
*Total*	92	162,520				

**FIGURE 5:**
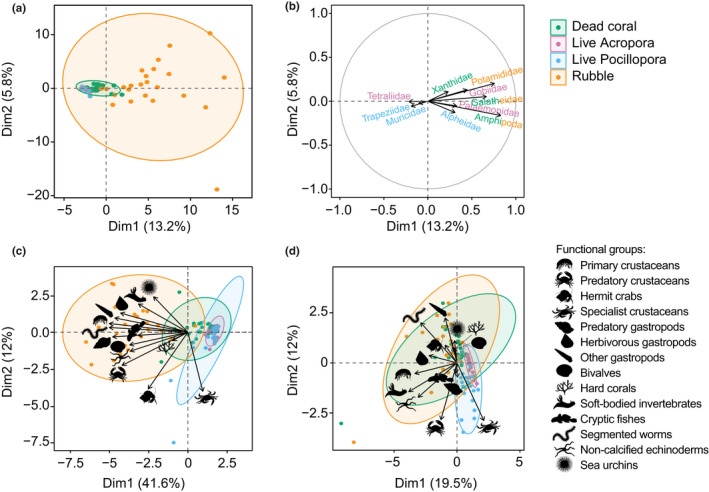
PCA of motile cryptofauna (a) density (m^−3^) per microhabitat by family, including (b) vector plot showing the strength and direction of the top‐three contributing families to the variation in species density within each habitat (*see* Table S5). Amphipoda and Galatheidae were key contributors in both dead coral and coral rubble habitat. PCA plots also shown for motile cryptofauna (c) density (m^−3^) and (d) biomass (g.m^−3^) by functional group.

Variation in community densities within and among habitats and sites was predominantly driven by dissimilarities in arthropods (SIMPER; Figures 4a and 5a,b; Tables S4 and S5). Specifically, Amphipoda and decapods from the Galatheidae, Palaemonidae and Xanthidae were the greatest contributors to dissimilarities in cryptic densities in dead coral and rubble compared to live coral (SIMPER; Figure 5b; Table S5). Tetraliidae and Trapeziidae (Decapoda) were the greatest contributors in *Acropora* (58% of the variation) and live *Pocillopora* (32% of the variation), respectively (Figure 5a,b; Table S5). Densities of palaemonid shrimps explained up to 10% of the variation among sites (Table S5). Of the remaining taxa, Potamididae and Muricidae (Gastropoda: Mollusca) were most prevalent in rubble and live *Pocillopora*, respectively (Figure 5a,b; Table S5). Ophiuroid (Echinodermata) densities were highest at NGK S, which drove dissimilarities between this and the remaining sites (Table S5). Densities of Gobiidae (Actinopterygii: Chordata) were also major contributors to differences among habitats and sites, particularly in live *Acropora* (26% of the variation) (Figure 5a,b; Table S5).

Cryptofauna biomass differed also across microhabitats and sites (Table 2, Table S4). Mean (±SE) biomass was greatest in live *Pocillopora* (29.0 ± 8.8 g.m^−3^), followed by rubble (27.6 ± 5.5 g.m^−3^), dead coral (16.1 ± 3.3 g.m^−3^) then live *Acropora* (5.7 ± 1.3 g.m^−3^) (Figure 4b). The disproportionate total biomass of fauna in live *Pocillopora* was largely due to muricid snails, but as for species densities, dissimilarities in average cryptic biomass were primarily driven by crustaceans (Figure 4b; Table S6). Diogenidae (Decapoda) represented the greatest total biomass, explaining up to 24% of the dissimilarities within habitats and 17% among sites (Table S6). In dead coral, variation in cryptic biomass was further explained by decapods of the Xanthidae (21% of the variation) and Galatheidae (12% of the variation) (Tables S4 and S6). Galatheidae, Palaemonidae and Gobiidae contributed most to dissimilarities in cryptofauna biomass in coral rubble (Tables S4 and S6). Tetraliid decapods were responsible for 73% of the variation in live *Acropora*, followed by Gobiidae (21% of the variation), while Trapeziidae contributed to 42% of the variation in live *Pocillopora*, along with Alpheidae (24% of the variation) and Muricidae (15% of the variation) (Tables S4 and S6).

Cryptofaunal size distributions revealed that individuals associated with live coral (*Acropora*, *Pocillopora*) had a broad size range with a greater representation of larger‐bodied individuals (>10 mm) (Figure 4e). Cryptofauna associated with dead coral and rubble showed pronounced peaks in smaller size classes (2–6 mm) (Figure 4e). Size distributions were significantly different among habitats (*p* < 0.001; Kruskal–Wallis test), but only when comparing live coral (either *Acropora* or *Pocillopora*) to either dead coral or rubble, not when comparing living or dead habitats to each other (Pairwise Wilcoxon tests).

### 3.4 Functional composition of the cryptofauna

A total of 14 functional groups were identified based on nine biological and ecological traits (Figure 2). Functional groups of cryptofauna (Figure 2) were closely related to phylogeny (Figure 3), likely because taxonomic data were pooled at the family level, which may confound interpretations of functional groups in some cases. However, our analysis delineated important functional roles above and beyond phylogeny, including habitat specialisation (e.g. coral‐associated crustaceans) and trophic guild (primary = detritivores and herbivores; secondary = predators) (Figure 2).

Within functional groups, cryptofauna densities varied across habitats and sites (Table 2, Table S4). Dissimilarities were most strongly influenced by primary, secondary and specialist crustaceans as well as fishes (SIMPER; Figures 4c and 5c; Tables S4 and S7). Specialist crustaceans explained 54% of the dissimilarity in live *Acropora* and 29% in live *Pocillopora* (Figure 5c; Tables S4 and S7). Primary and secondary crustaceans, gastropods and segmented worms were typically denser in dead coral and rubble (Figure 4c; Table S7). Primary crustacean density explained up to 13% and 24% of the dissimilarities within and among habitats, respectively (Tables S4 and S7). Fishes had greater densities in live *Acropora* (Figure 4c) explaining 29% of the dissimilarity in this microhabitat (Tables S4 and S7).

Overall, >80% of the dissimilarity in cryptofauna density and biomass in live *Acropora* could be explained by just two functional groups; specialist crustaceans and fishes (SIMPER; Table S4). Specialist crustaceans explained up to 68% of the variation in biomass within each habitat (Figures 4d and 5d; Table S4). Significant variation in biomass between dead and live coral was best explained by primary gastropods and primary crustaceans (~30% of the variation), which were weighted towards dead coral and rubble habitat (Figures 4d and 5d; Tables S4 and S8). Hermit crabs were also important components explaining 7%–15% and 5%–25% of the dissimilarities among habitats and sites, respectively (Table S8). The biomass of hermit crabs was disproportionate to their abundance and the remaining Arthropoda constituents (Figure 4c,d).

### 3.5 Predictors of cryptofauna functional group density and diversity

**FIGURE 6:**
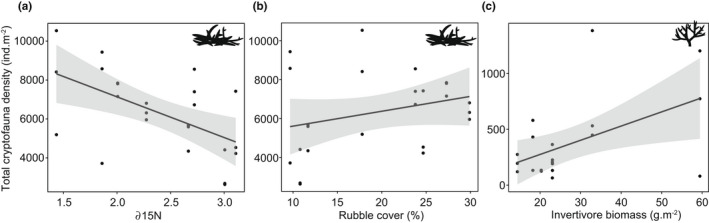
Relationships between the total density of cryptofauna in rubble with (a) increasing effluent exposure (*p* < 0.01) and (b) percent benthic cover of rubble (*p* = 0.04), and (c) in live *Acropora* with invertivore biomass (*p* = 0.05).

Water quality had mixed effects on the density of cryptofauna (Table 3). Primary crustaceans (*R* ^2^ = 0.34; *p* = 0.03) and bivalves (*R* ^2^ = 0.26; *p* = 0.02) exhibited a positive relationship with δ15^N^ in live *Acropora* while secondary gastropods (*R* ^2^ = 0.47; *p* = 0.046), other gastropods (*R* ^2^ = 0.41; *p* = 0.002) and bivalves (*R* ^2^ = 0.41; *p* < 0.001) in rubble were negatively impacted by δ15^N^ (Table 3). Overall, the total density of cryptofauna in rubble showed a negative relationship (*R* ^2^ = 0.42; *p* = 0.004) with δ15^N^ (Figure 6a). Species evenness in dead coral (*R* ^2^ = 0.17; *p* = 0.04) and rubble (*R* ^2^ = 0.19; *p* = 0.03) was positively associated with δ15^N^ (Figure S1).

**TABLE 3:** Summary of linear model results for bottom‐up (habitat cover, water quality) and top‐down (invertivore biomass) drivers on motile cryptofauna density by functional group. LA = live *Acropora*, LP = live *Pocillopora*, DC = dead coral, RU = rubble, green = positive relationship, red = negative relationship, blank cells denote no significant difference (*p* > 0.05)

	Benthic cover				Water quality (*δ15* ^*N*^)				Invertivore biomass			
	LA	LP	DC	RU	LA	LP	DC	RU	LA	LP	DC	RU
Primary crustaceans					+				+		+	
Hermit crabs						−						
Secondary crustaceans											+	−
Specialist crustaceans	+								+			
Primary gastropods											+	
Secondary gastropods				+				−	+			
Other gastropods	+							−				
Bivalves					+	+		−				
Hard coral												
Soft‐bodied invertebrates												
Fishes			+						+			
Segmented worms		+	+									
Non‐calcified echinoderms												
Sea urchins												
Total				+				−	+		+	

Site‐level benthic cover of rubble had a positive effect on the total density of cryptofauna occupying this microhabitat (*R* ^2^ = 0.42; *p* = 0.04) (Figure 6b). Positive associations were found between secondary gastropods in rubble (*R* ^2^ = 0.47; *p* = 0.001) and other gastropods in live *Acropora* (*R* ^2^ = 0.63; *p* < 0.001) based on the benthic cover of the respective microhabitat (Table 3). Specialist crustaceans were positively associated with the benthic cover of live *Acropora* (*R* ^2^ = 0.42; *p* = 0.002). Cryptobenthic fishes (*R* ^2^ = 0.20; *p* = 0.03) and segmented worms (*R* ^2^ = 0.32; *p* = 0.01) were positively associated with dead coral cover (Table 3). Species evenness showed a positive relationship with benthic cover of live *Acropora* (*R* ^2^ = 0.44; *p* = 0.01).

Invertivore biomass was not a strong predictor of cryptofauna density, yet significant relationships were found for certain functional groups (Table 3). Total cryptofauna density in live *Acropora* (*R* ^2^ = 0.24; *p* = 0.05; Figure 6c) and dead coral (*R* ^2^ = 0.34; *p* = 0.006) was positively associated with invertivorous reef fish biomass (Table 3). Specifically, primary crustaceans (*R* ^2^ = 0.34; *p* = 0.03), specialist crustaceans (*R* ^2^ = 0.42; *p* = 0.002), secondary gastropods (*R* ^2^ = 0.21; *p* = 0.04) and cryptobenthic reef fishes (*R* ^2^ = 0.24; *p* = 0.03) in live *Acropora* were positively associated with invertivore biomass (Table 3). In dead coral, primary (*R* ^2^ = 0.25; *p* = 0.025) and secondary crustaceans (*R* ^2^ = 0.33; *p* = 0.004) and primary gastropods (*R* ^2^ = 0.38; *p* = 0.001) were positively associated with invertivore biomass (Table 3). Secondary crustaceans in rubble were negatively associated with invertivore biomass (*R* ^2^ = 0.28; *p* = 0.02; Table 3). Species diversity in dead coral also showed a positive relationship with invertivore biomass (*R* ^2^ = 0.19; *p* = 0.04; Figure S1).

## 4 DISCUSSION

Cryptofauna diversity among all four microhabitats spanned ten phyla. Consistent with studies of terrestrial ecosystems (Wilson, 1992), biodiversity was dominated by invertebrates, which comprised 20 times the total number of families (diversity) and ~30 times the total number of individuals (density) identified compared to the vertebrates. Cryptofauna diversity was maximised when all four coral‐derived microhabitats were represented, given the occurrence of particular species in select microhabitats, such as trapeziid crabs, specialists of live *Pocillopora*. This highlights the importance of maintaining diverse habitat types on coral reefs in order to sustain high biodiversity.

Variations in cryptic community density, diversity and biomass were strongly influenced by microhabitat type, contributing to an emerging picture of microhabitats being a key predictor of the cryptofauna (Counsell et al., 2018; Enochs et al., 2011; Enochs & Manzello, 2012b; Fraser et al., 2021a; Fraser, Stuart‐Smith, et al., 2020; Pisapia et al., 2020; Takada et al., 2016; Wolfe, Desbiens, et al., 2020). While dead coral and rubble hosted a greater density and diversity of motile cryptofauna compared to living corals, this was dominated by a greater availability of small fauna. From a bottom‐up perspective, cryptic communities showed mixed responses to a water quality gradient from a sewage outfall to an oligotrophic reef. From a top‐down perspective, cryptofauna were generally unaffected by invertivore biomass. However, particular functional groups were positively associated with the biomass of invertivorous fishes, suggesting that predation pressure may result in higher turnover of lower trophic groups. It is likely that predation among the cryptofauna (Glynn & Enochs, 2011) and by nocturnal species are important drivers of cryptofauna community structure and productivity (Wolfe, Anthony, et al., 2020) but that cryptofauna are inherently resilient to top‐down control owing to their rapid population productivity (Coull, 1999; Wolfe et al., 2021). Yet, direct measures of cryptic population productivity in tropical reef systems, their availability as prey items, and trophic links to higher order predators remain to be quantified.

At a global scale, coral reefs have experienced widespread coral mortality due to an increased frequency and intensity of stressors (Cinner et al., 2016; Hughes, Anderson, et al., 2018). Underlying this is a shift in habitat condition, as live corals die and subsequently break down to rubble systems. Our analysis revealed striking diversity and abundance along this degradation continuum of coral‐derived habitats, as previously indicated for marine invertebrates (Enochs et al., 2011; Fraser et al., 2021a; Nelson et al., 2016) and cryptobenthic fishes (Ahmadia et al., 2012; Tornabene et al., 2013). Converse to the fish‐centric views that the high rugosity and complexity of corals enhances reef biodiversity (Coker et al., 2014; Graham, 2014; Graham et al., 2006), the dead coral and rubble matrix seems to provide the greatest complexity and security for smaller cryptic species (Enochs, 2012; Enochs & Manzello, 2012b; Hutchings, 1983; Wolfe et al., 2021). This is consistent with the theory that suggests small‐scale structural complexity supports the diversity and abundance of terrestrial invertebrates (Halaj et al., 2000; Hättenschwiler et al., 2005; Lassau et al., 2005; Wardhaugh et al., 2013). The sheer density and diversity of cryptofauna occupying dead coral and rubble evidences the importance of ‘degraded’ habitats in shaping coral reef communities, and that dead—not live—coral derives the greatest reef diversity (Enochs & Manzello, 2012b; Plaisance et al., 2011; Wolfe et al., 2021), as found for forest decay in terrestrial systems (Andringa et al., 2019).

Arthropoda (Crustacea) dominated samples in terms of density (55%) and biomass (58%), as broadly recognised for other reefs (Counsell et al., 2018; Gonzalez‐Gomez et al., 2018; Head et al., 2018; Klumpp et al., 1988; Kramer et al., 2014a; Pearman et al., 2018; Plaisance et al., 2011; Stella, Pratchett, et al., 2011). Interestingly, we document a reduction in size ranges along a profile of coral microhabitat degradation. Though not exclusive to the arthropods, this trend was largely influenced by high densities of smaller primary crustaceans (e.g. Amphipoda, Cumacea, Mysida, Isopoda) in dead coral and rubble, and larger coral‐associated decapods in live acroporids and pocilloporids (e.g. Trapeziidae, Tetraliidae). Diogenid hermit crabs were also important contributors to community dissimilarities, particularly in live *Pocillopora*, as for pebble crabs (Xanthidae) and snapping shrimp (Alpheidae) in coral rubble. These patterns in cryptic population structure have been observed across reef habitats and climatic zones, with important implications for reef trophodynamics (Fraser et al., 2021b; Fraser, Lefcheck, et al., 2020; Fraser, Stuart‐Smith, et al., 2020).

From a top‐down perspective, both macro‐ and micro‐invertebrates are critical elements of marine food webs (Glynn & Enochs, 2011; Opitz, 1993), with ~60% of invertivorous reef fish species on Australia's Great Barrier Reef suggested to feed predominantly on the Crustacea (Kramer et al., 2015). We predict that altered cryptic assemblages across current and future habitat degradation profiles will directly affect trophic links and food chain efficiency on coral reefs (Enochs & Manzello, 2012a), as shown for trophodynamics in the plankton (Dickman et al., 2008; Frederiksen et al., 2006). Modelled scenarios for degraded reefs suggest that increased invertebrate prey may support higher fisheries productivity, whereby the benefits from increased resource availability outweighs the costs of marginal declines in available coral refugia (Rogers, Blanchard, & Mumby, 2018). Yet, understanding how whole‐reef communities and fisheries productivity will respond over time requires explicit characterisation of the functional and trophic roles of cryptic species, as well as specific‐specific details on invertebrate‐invertivore trophic links across habitat types, to predict the likely winners and losers in future reef scenarios.

The high abundance of cryptofauna in dead coral and rubble is suggested to be promoted by an increase in the exploitable niches within the reef matrix (Enochs & Manzello, 2012b; Glynn & Enochs, 2011). In the context of ecosystem function, the predominance of cryptofauna in dead coral and rubble may be associated with the abundance of cryptoflora (e.g. epilithic and turf algae), sessile organisms (e.g. sponges and ascidians) and detritus (Enochs & Manzello, 2012b; Tews et al., 2004) typically associated with degraded and sheltered habitats (Borowitzka, 1981; Carpenter, 1986; Crossman et al., 2001; Wilson et al., 2003). This contrasts to the specialised symbioses between mucus‐feeding coral‐associated invertebrates (e.g. Tetraliidae, Trapeziidae) and their live coral hosts (Glynn, 1983; Stella, Pratchett, et al., 2011; Stimson, 1990). From a bottom‐up perspective, micro‐herbivores (e.g. amphipods, diogenid hermit crabs) can occupy specific ecological niches within the reef matrix as significant consumers of microfilamentous algae with a substantial influence on cryptobenthic community structure (Altman‐Kurosaki et al., 2018; Brawley & Adey, 1981; Klumpp et al., 1988; Klumpp & Pulfrich, 1989; Polunin & Klumpp, 1992). As posited for herbivorous fishes (Hughes et al., 2007; Mumby, 2006; Mumby et al., 2016), cryptic micro‐herbivores may be increasingly important in preventing the algal growth that can impede coral recovery (Altman‐Kurosaki et al., 2018). How this plays out in future reef scenarios is of interest, given the density and diversity of cryptofauna in dead coral and rubble (Enochs & Manzello, 2012b; Wolfe et al., 2021), and especially since small‐bodied organisms with short generation times (e.g. amphipods) may fare better in the climate change stakes (Kroeker et al., 2011; Milazzo et al., 2019; Sarmento et al., 2015; Timmers et al., 2021).

Live coral hosted the lowest cryptic species density and diversity, but the highest evenness coefficients. Many cryptic reef invertebrates—particularly crustaceans—have an obligate association with live coral, especially with *Acropora* and *Pocillopora* (Gonzalez‐Gomez et al., 2018; Stella, Pratchett, et al., 2011), where they utilise large amounts of coral mucus, and recycle detritus and organic matter (Glynn, 1983; Hutchings, 1983; Stimson, 1990). Not surprisingly, crustaceans of the Trapeziidae and Tetraliidae (Decapoda) were primarily associated with these coral genera in our surveys and were seldom found in dead coral and coral rubble. In a broad review of coral‐associated invertebrates, 56% (487 of 869) were found to be obligates to their live coral host (Stella, Pratchett, et al., 2011). As their survival is inextricably linked to that of their host, obligate coral associates are considered particularly vulnerable to changes in live coral condition (Caley et al., 2001; Stella, Munday, & Jones, 2011; Stella, Pratchett, et al., 2011). Their populations are also likely constrained by habitat abundance, with high intra‐specific competition for suitable habitats (Enochs, 2012; Head et al., 2015; Nelson et al., 2016; Stella et al., 2014), and for disturbances to enhance some cryptofaunal populations (Kroeker et al., 2011; Milazzo et al., 2019; Moran & Reaka‐Kudla, 1991; Sarmento et al., 2015; Timmers et al., 2021). Further work is needed to understand the vulnerabilities of cryptofauna (especially obligate associates) to anthropogenic stressors, in light of recent mass coral bleaching and mortality events (Heron et al., 2016; Hughes, Kerry, et al., 2018).

The water quality gradient across our study sites had mixed effects on cryptofauna. Densities of palaemonid shrimp and *Drupella* (Mollusca) were highest at sites closer to the sewage outfall, while correlations between total cryptofauna density and sewage exposure in rubble were negative. Positive associations were found between water quality and species evenness in dead coral and rubble. However, the nitrogen concentrations used as a proxy for water quality here (as in Roff et al., 2019) are below levels suggested to be high on other coral reefs influenced by sewage outfalls (e.g. Redding et al., 2013; Risk et al., 2009), likely due to low water residence times in Malakal Bay (Birkeland et al., 1976). While some relationships between cryptofauna and sewage exposure were evident, it is likely that the values of δ15^N^ used were below thresholds for significant ecological consequence.

The observed bottom‐up and top‐down trends in cryptic assemblages are somewhat surprising given the incredible uniqueness typical of coral reef cryptofauna across minute spatial scales (Brandl et al., 2018; Carvalho et al., 2019; Hutchings, 1983; Hutchings & Kupriyanova, 2015; Middelfart et al., 2016; Plaisance et al., 2009; Plaisance et al., 2011; Takada et al., 2016). Although nocturnal invertivores were excluded owing to daytime surveys, and could represent a major component of invertivorous fish assemblages (Wolfe, Anthony, et al., 2020), the positive associations between some functional groups of cryptofauna and invertivorous reef fish biomass highlight the importance of cryptic species to coral reef food webs (Glynn & Enochs, 2011). Yet, invertivore biomass did not influence cryptofauna in community‐level analyses (i.e. PERMANOVA), which highlights the resilience of cryptofauna communities to predator‐driven depletion (Coull, 1990), the importance of identifying specialist groups, and the intricacies of invertivory pathways. Given the high degrees of diversity and specialisation among invertivorous coral reef fishes (Bellwood et al., 2010; Hiatt & Strasburg, 1960; Hobson, 1974; Kramer et al., 2015; Williams & Hatcher, 1983; Wolfe, Anthony, et al., 2020), invertivore biomass may not be a suitable indicator of trophic pressures on cryptofauna communities. While cryptofauna densities can be regulated by predation (e.g. cryptobenthic fishes; Brandl et al., 2018, 2019; Goatley et al., 2017; Goatley & Brandl, 2017), their rates of productivity (i.e. turnover) and availability as prey to higher order consumers require specific attention in tropical reef ecosystems. Our data give insight into the potential contribution of cryptofauna to reef trophic structure originating from different coral‐derived microhabitats. Quantifying cryptic population productivity and energy transfers within the reef matrix, and beyond, is essential to the characterisation of tractable trophodynamic and ecosystem‐based fisheries models, and in predicting future reef resilience (Brandl et al., 2018; Kramer et al., 2015; Pauly et al., 1998; Smith et al., 2011; Wolfe et al., 2021).

The high functional diversity of cryptofauna may confer higher resilience of coral reef ecosystems through the maintenance of key ecological functions after disturbance. While the highest level of species diversity is upheld by all microhabitats combined, the greater density and biomass of cryptofauna in dead coral and rubble may trigger compensatory food webs as coral reefs deteriorate through the maintenance of biodiversity (Altman‐Kurosaki et al., 2018; Enochs & Manzello, 2012b) and redistribution of the of prey available to fishes (Fraser et al., 2021a, 2021b; Fraser, Lefcheck, et al., 2020). However, we acknowledge that this may not necessarily translate to greater fisheries productivity. There are several reasons for this. First, the direct effects of fish predation on the turnover of cryptofauna have not been quantified, and higher cryptofauna biomass in degraded habitats could imply that fish predation rates are lower, which limits the scope for uptake in fish‐based trophic pathways. Second, validated models of reef fisheries productivity find that marked loss of reef habitat complexity (i.e. transition from intact coral colonies to coral rubble) reduces productivity because of the lack of refugia for fishes, though fisheries productivity can be maintained and even elevated in dead coral and rubble reefs that retain some structure (Morais et al., 2020; Rogers et al., 2014; Rogers, Blanchard, & Mumby, 2018; Rogers, Blanchard, Newman, et al., 2018). It is essential that these nuances in trophic and disturbance ecology are addressed in future research.

There is now a critical need to determine how cryptic biodiversity influences food webs on current and future coral reefs. Disturbances that adversely affect live coral will play vital roles in the redesign of coral reef trophodynamics and ecosystem function. Results presented here highlight the importance of coral rubble in the total and long‐term productivity of coral reef ecosystems, potentially supporting the food web after a disturbance has impacted healthy corals (Wolfe et al., 2021). Given the increasing frequency and severity of disturbances impacting coral reefs and causing global declines in live coral (Hughes, Anderson, et al., 2018), there is an emerging need to move beyond live coral cover as an indicator of ecosystem health and to quantify the contribution of habitats other than live coral to ecosystem productivity. This may include empirical characterisation of food webs and energy transfers derived from dead coral and rubble to improve our ability to predict productivity and trophodynamic outcomes under future reefs scenarios.

## AUTHOR CONTRIBUTIONS

Peter J. Mumby, Jessica Stella, Alice Rogers and George Roff designed the study; Benthic surveys were completed by Jessica Stella, and images were analysed by Alice Rogers; Cryptofauna collection and identification was completed by Jessica Stella; Reef fish community composition was assessed by Mark Priest and Jessica Stella; Kennedy Wolfe, Jessica Stella and George Roff managed data handling, synthesis and analysis; Jessica Stella and Kennedy Wolfe wrote the manuscript, and all authors contributed to subsequent drafts.

## CONFLICT OF INTEREST

The authors declare no conflict of interest.

## Supporting information


Appendix S1

## Data Availability

Data pertaining to this study have been made available at GitHub (Zenodo) https://doi.org/10.5281/zenodo.7039606 (Stella et al., 2022). No novel code was created or used.
