# Supplementary material for: Functional and phylogenetic responses of motile cryptofauna to habitat degradation
Source: J Anim Ecol. 2022 Sep 11;91(11):2203–19. doi: 10.1111/1365-2656.13809 (PMC9826372; doi:10.1111/1365-2656.13809)
Supplement: Supplementary file 1 — Appendix S1 [file JANE-91-2203-s001.docx]

Supplementary material - Journal of Animal Ecology

***Functional and phylogenetic responses of motile cryptofauna to habitat degradation***

Jessica S. Stella, Kennedy Wolfe*, George Roff, Alice Rogers, Mark Priest, Yimnang Golbuu, Peter J. Mumby


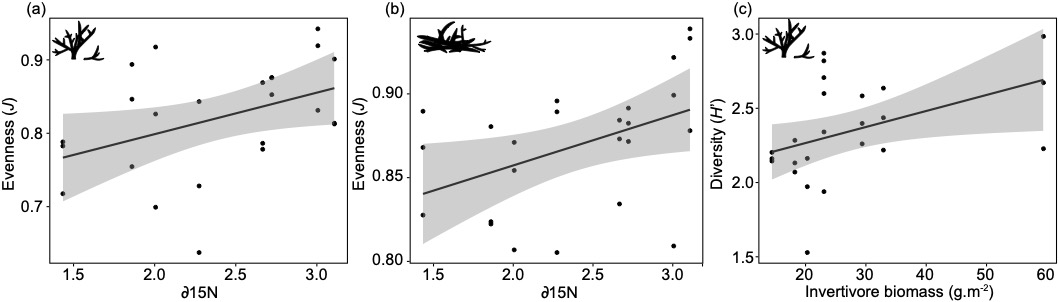


**Fig. S1:** Motile cryptofauna diversity (*H’*; Shannon-Weaver index) and evenness (J; Pielou’s coefficient) in (a.) dead coral and (b.) rubble against water quality (∂15N), and in (c.) dead coral against invertivore biomass.

**Table S1:** List of all invertivorous coral reef fishes encountered across eight survey sites on the east coast of Palau. Regression coefficients (a, b) were used to calculate length-weight data using Fishbase (Froese and Pauly 2019); asterisk denotes values for whole genus due to limited species-specific data.

| **Family** | **Species** | **a** | **b** |
| --- | --- | --- | --- |
| Balistidae | *Balistapus undulatus* | 0.0309 | 3.11 |
|  | *Sufflamen bursa* | 0.0240* | 3.02* |
|  | *chrysopterus* | 0.0240* | 3.02* |
| Chaetodontidae | *Chaetodon bennetti* | 0.0384 | 2.89 |
|  | *citrinellus* | 0.0380 | 2.81 |
|  | *ephippium* | 0.0229 | 3.05 |
|  | *kleinii* | 0.0447 | 2.96 |
|  | *lineolatus* | 0.0693 | 2.62 |
|  | *lunulatus* | 0.0288* | 3.01* |
|  | *melannotus* | 0.0316 | 2.98 |
|  | *octofasciatus* | 0.0288* | 3.01* |
|  | *ornatissimus* | 0.0288* | 3.01* |
|  | *punctatofasciatus* | 0.0288* | 3.01* |
|  | *rafflesii* | 0.0288* | 3.01* |
|  | *reticulatus* | 0.0288* | 3.01* |
|  | *semeion* | 0.0288* | 3.01* |
|  | *speculum* | 0.0664 | 2.69 |
|  | *ulietensis* | 0.0311 | 2.87 |
|  | *unimaculatus* | 0.0533 | 2.83 |
|  | *vagabundus* | 0.0278 | 2.97 |
|  | *Forcipiger longirostris* | 0.0188* | 3.01* |
|  | *Heniochus chrysostomus* | 0.0145 | 3.32 |
|  | *monoceros* | 0.0195 | 3.17 |
|  | *varius* | 0.0195* | 3.19* |
| Haemulidae | *Plectorhinchus chaetodonoides* | 0.0234 | 3.02 |
| Holocentridae | *Neoniphon sammara* | 0.0162 | 3.03 |
|  | *Sargocentron caudimaculatum* | 0.0391 | 2.94 |
|  | *praslin* | 0.0224* | 2.96* |
| Labridae | *Anampses meleagrides* | 0.0171* | 3.00* |
|  | *twisti* | 0.0171* | 3.00* |
|  | *Bodianus axillaris* | 0.0171* | 3.00* |
|  | *Cheilinus fasciatus* | 0.0200* | 3.00* |
|  | *sp.* | 0.0200* | 3.00* |
|  | *undulatus* | 0.0151 | 3.06 |
|  | *Coris batuensis* | 0.0068* | 3.15* |
|  | *gaimard* | 0.0068* | 3.15* |
|  | *Diproctacanthus xanthurus* | 0.0076 | 3.105 |
|  | *Epibulus insidiator* | 0.0339 | 2.91 |
|  | *Gomphosus varius* | 0.0049 | 2.93 |
|  | *Halichoeres chloropterus* | 0.0160 | 2.87 |
|  | *hortulanus* | 0.0119 | 3.06 |
|  | *leucurus* | 0.0107* | 3.13* |
|  | *marginatus* | 0.0132 | 3.14 |
|  | *melanurus* | 0.0105 | 3.33 |
|  | *Hemigymnus fasciatus* | 0.0234* | 2.98* |
|  | *melapterus* | 0.0237 | 2.97 |
|  | *Labrichthys unilineatus* | 0.0204 | 3.00 |
|  | *Macropharyngodon melegris* | 0.0207 | 3.00 |
|  | *Monotaxis grandoculus* | 0.0355 | 2.89 |
|  | *Oxycheilinus digrammus* | 0.0389 | 2.85 |
|  | *sp.* | 0.0363* | 2.69* |
|  | *unifasciatus* | 0.0363* | 2.69* |
|  | *Pseudocheilinus hexatania* | 0.0176* | 3.16* |
|  | *Stethojulis bandanensis* | 0.0219 | 2.76 |
|  | *Thalassoma amblycephalum* | 0.0155* | 2.95* |
|  | *hardwicke* | 0.0135 | 3.04 |
|  | *lunare* | 0.0240 | 2.82 |
| Lutjanidae | *Lutjanus gibbus* | 0.0191 | 3.00 |
|  | *semicinctus* | 0.0040 | 3.43 |
| Malacanthidae | *Malacanthus latovittatus* | 0.0158* | 2.78* |
| Monacanthidae | *Cantherhines dumerilii* | 0.0398* | 2.78* |
| Mullidae | *Parupeneus barberinus* | 0.0162 | 2.99 |
|  | *cyclostomus* | 0.0136 | 3.11 |
|  | *multifasciatus* | 0.0151 | 3.10 |
| Nemipteridae | *Scolopsis bilineata* | 0.0145 | 3.16 |
|  | *margaritifer* | 0.0333 | 2.97 |
| Pomacanthidae | *Chaetodontoplus mesoleucus* | 0.0437* | 2.82* |
| Serranidae | *Variola louti* | 0.0135 | 3.05 |
| Synodontidae | *Synodus sp.* | 0.0042* | 3.25* |
| Tetraodontidae | *Canthigaster solandri* | 0.0299 | 2.98 |
|  |  |  |  |

**Table S2:** Complete list of motile cryptofauna identified.

| **Phylum** | **Class** | **Order** | **Family** | **Common name** |
| --- | --- | --- | --- | --- |
| Cnidaria | Anthozoa | Actiniaria |  | Sea anemone |
|  |  | Scleractinia |  | Stony coral |
| Platyhelminthes | Rhadbitophora | Polycladida | Pseudocerotidae | Bedford's flatworm |
|  |  |  |  | *Unidentified* |
| Nemertea |  |  |  |  |
| Nematoda |  |  |  |  |
| Sipuncula | Phascolosomatidea | Phascolosomatida | Phascolosomatidae | Peanut worm |
|  | Sipunculidea | Golfingiida | Golfingiidae |  |
|  |  |  |  | *Unidentified* |
| Annelida | Clitellata | Oligochaeta |  |  |
|  | Polychaeta | Aciculata | Amphinomidae | Bristle worm |
|  |  | Echiuroidea | Bonelliidae | Spoon worm |
|  |  | Eunicida | Eunicidae | Bobbit worm |
|  |  | Phyllodocida | Hesionidae | Bristle worm |
|  |  |  | Nereididae | Ragworm |
|  |  |  | Phyllodocidae | Paddleworm |
|  |  |  | Polynoidae | Scale worm |
|  |  |  | Syllidae | Syllid worm |
|  |  | Terebellida | Cirratulidae | Thread worm |
|  |  |  |  | Bristle worm |
|  |  |  |  | *Unidentified* |
| Mollusca | Bivalvia | Cardiida | Cardiidae | Cockle |
|  |  | Limida | Limidae | File shell |
|  |  | Mytilida | Mytilidae | Saltwater mussel |
|  |  | Ostreida | Pteriidae | Feather oyster |
|  |  | Pectinida | Pectinidae | Scallop |
|  |  | Venerida | Veneridae | Clam |
|  |  |  |  | *Unidentified* |
|  | Gastropoda | Aplysiida | Aplysiidae | Sea hare |
|  |  | Caenogastropoda | Dialidae | Dialid snail |
|  |  |  | Epitoniidae | Wentletrap |
|  |  |  | Potamididae | Horn snail |
|  |  |  | Terebridae | Auger snail |
|  |  |  | Triphoridae | Left-handed snail |
|  |  | Cephalaspidea | Aglajidae | Headshield slug |
|  |  |  | Aplustridae | Bubble snail |
|  |  |  | Bullidae | Bubble snail |
|  |  |  | Haminoeidae | Bubble snail |
|  |  | Lepetellida | Fissurellidae | Keyhole limpet |
|  |  |  | Haliotidae | Abalone |
|  |  | Littorinimorpha | Cypraeidae | Cowry snail |
|  |  |  | Littorinidae | Periwinkle |
|  |  |  | Naticidae | Moon snail |
|  |  |  | Ovulidae | Egg cowry |
|  |  |  | Rissoinidae | Risso snail |
|  |  |  | Strombidae | Conch snail |
|  |  |  | Triviidae | Allied cowry |
|  |  |  | Vermetidae | Worm snail |
|  |  | Neogastropoda | Buccinidae | Whelk |
|  |  |  | Cancellariidae | Nutmeg snail |
|  |  |  | Columbellidae | Dove snail |
|  |  |  | Conidae | Cone snail |
|  |  |  | Costellariidae | Ribbed miters |
|  |  |  | Fasciolariidae | Tulip snail |
|  |  |  | Mitridae | Mitre shell |
|  |  |  | Muricidae | Murex snail |
|  |  |  | Olividae | Olive snail |
|  |  |  | Turbinellidae | Vase snail |
|  |  |  | Turridae |  |
|  |  |  | Volutidae | Volutes |
|  |  | Nudibranchia |  | Nudibranch |
|  |  | Patellogastropoda | Patellidae | Limpet |
|  |  | Pleurobranchida | Pleurobranchidae | Side-gill slug |
|  |  | Pylopulmonata | Pyramidellidae | Pyramid snail |
|  |  | Sacoglossa | Plakobranchidae | Sea slug |
|  |  | Trochida | Liotiidae | Liotia |
|  |  |  | Phasianellidae | Pheasant snail |
|  |  |  | Trochidae | Top-snail |
|  |  |  | Turbinidae | Turban snail |
|  |  |  |  | *Unidentified mollusc* |
|  |  |  |  | *Unidentified Euthyneura* |
|  | Polyplacophora | Chitonida | Chitonidiae | Chiton |
| Arthropoda | Malacostraca | Amphipoda | Caprellidae | Skeleton shrimp |
| (Crustacea) |  |  |  | *Unidentified* |
|  |  | Cumacea |  | Hooded shrimp |
|  |  | Decapoda | Alpheidae | Snapping shrimp |
|  |  |  | Brachyura |  |
|  |  |  | Diogenidae | Hermit crab |
|  |  |  | Dromiidae | Sponge crab |
|  |  |  | Epialtidae | Spider crab |
|  |  |  | Galatheidae | Squat lobster |
|  |  |  | Hippolytidae | Anemone shrimp |
|  |  |  | Majidae | Spider crab |
|  |  |  | Paguridae | Hermit crab |
|  |  |  | Palaemonidae | Palaemonid shrimp |
|  |  |  | Penaeidae | Penaeid shrimp |
|  |  |  | Pilumnidae | Hairy crabs |
|  |  |  | Porcellanidae | Porcelain crab |
|  |  |  | Portunidae | Swimming crab |
|  |  |  | Tetraliidae |  |
|  |  |  | Trapeziidae | Coral crab |
|  |  |  | Xanthidae | Pebble crab |
|  |  | Isopoda | Anthuridae |  |
|  |  |  | Asellidae |  |
|  |  |  | Joeropsididae |  |
|  |  |  | Paranthuridae |  |
|  |  |  | Stenetriidae |  |
|  |  |  |  | *Unidentified* |
|  |  | Mysida |  | Opossum shrimp |
|  |  | Stomatopoda | Gonodactylidae | Mantis shrimp |
|  |  |  |  | *Unidentified* |
|  |  | Tanaidacea | Paratanaidae | Tanaids |
|  |  |  |  | *Unidentified* |
|  | Ostracoda |  |  | Seed shrimp |
|  | Pycnogonida |  |  | Sea spider |
| Echinodermata | Asteroidea | Valvatida | Asterinidae | Seastar |
|  |  |  | Ophidiasteridae |  |
|  | Crinoidea |  |  | Feather star |
|  | Echinoidea | Camarodonta | Toxopneustidae |  |
|  |  | Cidaroida | Cidaridae | Pencil urchin |
|  |  | Diadematoida | Diadematidae | Diademid urchin |
|  | Ophiuroidea | Amphilepidida | Ophiactidae | Brittle star |
|  |  |  | Ophiotrichidae |  |
|  |  | Ophiacanthida | Ophiocomidae |  |
|  |  |  | Ophiomyxidae |  |
|  |  |  |  | *Unidentified* |
| Chordata | Actinopterygii | Perciformes | Blenniidae | Blennies |
|  |  |  | Gobiidae | Gobies |
|  |  |  | Labridae | Wrasse |
|  |  |  | Pomacentridae | Damselfish |
|  |  | Scorpaeniformes | Scorpaenidae | Scorpionfish |
|  |  | Tetraodontiformes | Monacanthidae | Filefish |

**Table S3:** Summary statistics (PERMANOVA) for differences in benthic composition (live *Acropora*, live *Pocillopora*, dead branching coral and coral rubble), and invertivorous reef fish density (ind.m^-2^) and biomass (g.m^-2^) among the eight survey sites on the east coast of Palau. Significant values in bold.

| **Source** | **df** | **SS** | **MS** | ***p* value** | **Unique perms** |
| --- | --- | --- | --- | --- | --- |
| ***Benthic composition*** |  |  |  |  |  |
| *Site* | 7 | 44959 | 6422.7 | **0.001** | 998 |
| *Res* | 208 | 107540 | 517.0 |  |  |
| *Total* | 215 | 152500 |  |  |  |
| ***Invertivore density*** |  |  |  |  |  |
| *Site* | 7 | 30.4 | 4.34 | **<0.001** | 997 |
| *Res* | 16 | 30.2 | 1.89 |  |  |
| *Total* | 23 | 60.6 |  |  |  |
| ***Invertivore biomass*** |  |  |  |  |  |
| *Site* | 7 | 448.0 | 64.0 | **0.01** | 996 |
| *Res* | 16 | 625.1 | 39.1 |  |  |
| *Total* | 23 | 1073.1 |  |  |  |

**Table S4:** Results of SIMPER analyses of motile cryptofauna that contributed most strongly to similarities in density or biomass by Family (taxonomy) or functional group (function) within each survey site and microhabitat. Data are listed in order of their contribution to similarities (top six or top 90%).

|  | **Taxonomy** | | **Function** | |  |  | **Taxonomy** | | **Function** | |  |
| --- | --- | --- | --- | --- | --- | --- | --- | --- | --- | --- | --- |
| **Site** | **Density** | **Biomass** | **Density** | **Biomass** |  | **Microhabitat** | **Density** | **Biomass** | **Density** | **Biomass** | |
| **SEW 1** | *49.3%* | *43.6%* | *72.1%* | *60.0%* |  | ***Acropora*** | *45.9%* | *47.2%* | *59.3%* | *62.1%* | |
|  | Palaemonidae | Trapeziidae | PC | SPC |  |  | Tetraliidae | Tetraliidae | SPC | SPC | |
|  | Alpheidae | Tetraliidae | SPC | PC |  |  | Gobiidae | Gobiidae | FISH | FISH | |
|  | Trapeziidae | Alpheidae | NCE | FISH |  |  | Palaemonidae |  | PC |  | |
|  | Tetraliidae | Palaemonidae | SG | SG |  |  |  |  |  |  | |
|  | Xanthidae | Gobiidae | FISH | NCE |  |  |  |  |  |  | |
|  | Potamididae | Xanthidae | SC | BIV |  |  |  |  |  |  | |
| **SEW 2** | *55.7%* | *51.7%* | *67.7%* | *64.9%* |  | ***Pocillopora*** | 52.4% | *56.9%* | *65.5%* | *63.0%* | |
|  | Palaemonidae | Tetraliidae | PC | SPC |  |  | Trapeziidae | Trapeziidae | SPC | SPC | |
|  | Tetraliidae | Trapeziidae | SPC | PC |  |  | Alpheidae | Alpheidae | SC | SC | |
|  | Trapeziidae | Alpheidae | SG | SG |  |  | Muricidae | Muricidae | PC | SG | |
|  | Alpheidae | Palaemonidae | SC | SC |  |  | Palaemonidae | Diogenidae | SG | HC | |
|  | Xanthidae | Xanthidae | SW | PG |  |  | Diogenidae | Palaemonidae | NCE | NCE | |
|  | Galatheidae | Galatheidae | FISH | FISH |  |  | Ophiactidae |  | HC |  | |
| **SEW 3** | *43.1%* | *39.7%* | *64.3%* | *62.7%* |  | **Dead coral** | *44.6%* | *34.9%* | *70.1%* | *56.0%* | |
|  | Trapeziidae | Trapeziidae | SPC | SPC |  |  | Xanthidae | Diogenidae | PC | PC | |
|  | Gobiidae | Gobiidae | PC | PC |  |  | Amphipoda | Xanthidae | SW | HC | |
|  | Tetraliidae | Tetraliidae | SG | SG |  |  | Galatheidae | Galatheidae | SG | SG | |
|  | Palaemonidae | Diogenidae | SC | HC |  |  | Polychaeta | Amphipoda | OG | NCE | |
|  | Alpheidae | Galatheidae | FISH | FISH |  |  | Potamididae | Fasciolariidae | SC | OG | |
|  | Galatheidae | Alpheidae | OG | OG |  |  | Diogenidae | Potamididae | NCE | SW | |
| **SEW 4** | *47.2%* | *42.9%* | *69.6%* | *59.3%* |  | **Rubble** | *55.7%* | *40.8%* | *84.5%* | *65.5%* | |
|  | Trapeziidae | Muricidae | PC | SPC |  |  | Galatheidae | Galatheidae | PC | PC | |
|  | Gobiidae | Trapeziidae | SPC | SG |  |  | Potamididae | Gobiidae | SG | PG | |
|  | Alpheidae | Alpheidae | SG | PC |  |  | Amphipoda | Palaemonidae | OG | SG | |
|  | Muricidae | Gobiidae | FISH | SC |  |  | Palaemonidae | Potamididae | SW | FISH | |
|  | Palaemonidae | Tetraliidae | SC | FISH |  |  | Gobiidae | Haliotidae | PG | OG | |
|  | Galatheidae | Galatheidae | OG | PG |  |  | Polychaeta | Fasciolariidae | SC | SC | |
| **NGK S** | *45.2%* | *37.2%* | *77.8%* | *65.0%* |  |  |  |  |  |  | |
|  | Alpheidae | Alpheidae | SC | NCE |  |  |  |  |  |  | |
|  | Trapeziidae | Trapeziidae | NCE | PC |  | Functional | PC | Primary crustaceans  Secondary crustaceans  Hermit crabs  Specialist crustaceans  Primary gastropods  Secondary gastropods  Other gastropods  Bivalves  Hard coral  Soft-bodied invert  Fishes  Segmented worms | |  | |
|  | Amphipoda | Xanthidae | PC | SC |  | Groups: | SC |  |  |  | |
|  | Ophiuroidea | Diogenidae | SG | SPC |  |  | HC |  |  |  | |
|  | Xanthidae | Galatheidae | SW | HC |  |  | SPC |  |  |  | |
|  | Palaemonidae | Amphipoda | SPC | SG |  |  | PG |  |  |  | |
| **NGK M** | *57.2%* | *53.4%* | *75.0%* | *67.2%* |  |  | SG |  |  |  | |
|  | Tetraliidae | Diogenidae | SPC | SPC |  |  | OG |  |  |  | |
|  | Gobiidae | Tetraliidae | FISH | HC |  |  | BIV |  |  |  | |
|  | Alpheidae | Gobiidae | PC | PC |  |  | COR |  |  |  | |
|  | Diogenidae | Alpheidae | SC | FISH |  |  | SBI |  |  |  | |
|  | Amphipoda | Trapeziidae | NCE | SC |  |  | FISH |  |  |  | |
|  | Trapeziidae | Galatheidae | SG | SG |  |  | SW |  |  |  | |
| **NGK N** | *52.6%* | *48.4%* | *70.0%* | *58.9%* |  |  | NCE | Non-calcified echino |  |  | |
|  | Gobiidae | Tetraliidae | SPC | SPC |  |  | SU | Sea urchins |  |  | |
|  | Tetraliidae | Diogenidae | PC | HC |  |  |  |  |  |  | |
|  | Trapeziidae | Gobiidae | FISH | PC |  |  |  |  |  |  | |
|  | Alpheidae | Trapeziidae | SC | FISH |  |  |  |  |  |  | |
|  | Muricidae | Alpheidae | SG | SG |  |  |  |  |  |  | |
|  | Diogenidae | Muricidae | HC | SC |  |  |  |  |  |  | |
| **SDO** | *46.9%* | *40.0%* | *68.0%* | *55.9%* |  |  |  |  |  |  | |
|  | Tetraliidae | Tetraliidae | SPC | SPC |  |  |  |  |  |  | |
|  | Trapeziidae | Trapeziidae | PC | PC |  |  |  |  |  |  | |
|  | Galatheidae | Galatheidae | FISH | SG |  |  |  |  |  |  | |
|  | Polychaeta | Diogenidae | SG | FISH |  |  |  |  |  |  | |
|  | Palaemonidae | Palaemonidae | SC | PG |  |  |  |  |  |  | |
|  | Alpheidae | Muricidae | SW | HC |  |  |  |  |  |  | |

**Table S5:** Results of SIMPER analysis of motile cryptofauna (by Family) that contributed most strongly to dissimilarities in density between locations and microhabitats. Families are listed in order of their contribution (top six) to dissimilarities. Grey cells show t- and p-values from Pairwise tests (PERMANOVA) with significant values in bold (df = 13–16, unique perms = 997–999); categories to the left of the cell were greater in the factor labelled by row, while categories to the right were greater in the factor labelled by column.

|  | **SEW 1** |  |  | ***Acropora*** | ***Pocillopora*** | **Dead coral** |  |
| --- | --- | --- | --- | --- | --- | --- | --- |
| **SEW 2** | 1.42, **0.044** |  |  | 6.64, **0.001** | 7.50, **0.001** | 3.00, **0.001** | **Rubble** |
|  | *53.7%* |  |  | *90.2%* | *86.3%* | *61.4%* |  |
|  | Gobiidae |  |  | Galatheidae | Galatheidae | Hippolytidae |  |
|  | Ophiuroidea |  |  | Potamididae | Potamididae | Tanaidacea |  |
|  | Palaemonidae |  |  | Amphipoda | Amphipoda | Ophiuroidea |  |
|  | Muricidae |  |  | Isopoda | Trapeziidae | Haliotidae |  |
|  | Limidae |  |  | Polychaeta | Polychaeta | Palaemonidae |  |
|  | Ophiactidae | **SEW 2** |  | Alpheidae | Isopoda | Gobiidae |  |
| **SEW 3** | 1.39, 0.052 | 1.39, **0.050** |  |  | 5.57, **0.001** | 5.47, **0.001** | ***Acropora*** |
|  | *59.4%* | *54.4%* |  |  | *81.8%* | *89.7%* |  |
|  | Gobiidae | Palaemonidae |  |  | Trapeziidae | Xanthidae |  |
|  | Palaemonidae | Gobiidae |  |  | Tetraliidae | Amphipoda |  |
|  | Ophiuroidea | Limidae |  |  | Alpheidae | Galatheidae |  |
|  | Ophiactidae | Alpheidae |  |  | Muricidae | Tetraliidae |  |
|  | Muricidae | Ophiocomidae |  |  | Gobiidae | Polychaeta |  |
|  | Diogenidae | Diogenidae | **SEW 3** |  | Palaemonidae | Potamididae |  |
| **SEW 4** | 1.35, **0.036** | 1.41, **0.036** | 0.87, 0.699 |  |  | 5.84, **0.001** | ***Pocillopora*** |
|  | *58.4%* | *53.8%* | *55.4%* |  |  | *82.0%* |  |
|  | Palaemonidae | Gobiidae | Palaemonidae |  |  | Amphipoda |  |
|  | Ophiuroidea | Palaemonidae | Ophiactidae |  |  | Trapeziidae |  |
|  | Ophiactidae | Limidae | Alpheidae |  |  | Xanthidae |  |
|  | Gobiidae | Galatheidae | Gobiidae |  |  | Galatheidae |  |
|  | Galatheidae | Alpheidae | Diogenidae |  |  | Potamididae |  |
|  | Pectinidae | Ophiactidae | Galatheidae | **SEW 4** |  | Polychaeta |  |
| **NGK S** | 1.79, **0.004** | 2.06, **0.001** | 1.45, **0.024** | 1.41, **0.018** |  |  |  |
|  | *60.2%* | *56.0%* | *57.3%* | *55.1%* |  |  |  |
|  | Palaemonidae | Muricidae | Ophiomyxidae | Muricidae |  |  |  |
|  | Ophiuroidea | Ophiuroidea | Ophiactidae | Ophiuroidea |  |  |  |
|  | Ophiomyxidae | Ophiactidae | Palaemonidae | Ophiomyxidae |  |  |  |
|  | Muricidae | Palaemonidae | Alpheidae | Ophiactidae |  |  |  |
|  | Pectinidae | Gobiidae | Muricidae | Palaemonidae |  |  |  |
|  | Ophiactidae | Ophiocomidae | Ophiuroidea | Scorpaenidae | **NGK S** |  |  |
| **NGK M** | 1.88, **0.001** | 1.90, **0.002** | 1.01, 0.408 | 1.28, 0.107 | 1.73, **0.001** |  |  |
|  | *56.2%* | *52.0%* | *50.3%* | *53.8%* | *52.7%* |  |  |
|  | Diogenidae | Gobiidae | Palaemonidae | Diogenidae | Diogenidae |  |  |
|  | Palaemonidae | Diogenidae | Gobiidae | Palaemonidae | Muricidae |  |  |
|  | Gobiidae | Palaemonidae | Tetraliidae | Galatheidae | Ophiactidae |  |  |
|  | Ophiuroidea | Limidae | Diogenidae | Ophiactidae | Ophiuroidea |  |  |
|  | Ophiactidae | Xanthidae | Trapeziidae | Gobiidae | Palaemonidae |  |  |
|  | Mytilidae | Alpheidae | Ophiocomidae | Tetraliidae | Ophiomyxidae | **NGK M** |  |
| **NGK N** | 1.89, **0.001** | 2.07, **0.001** | 1.50, **0.019** | 1.54, **0.006** | 1.78, **0.001** | 1.52, **0.013** |  |
|  | *59.7%* | *55.0%* | *56.4%* | *56.4%* | *53.8%* | *49.2%* |  |
|  | Palaemonidae | Palaemonidae | Palaemonidae | Palaemonidae | Muricidae | Diogenidae |  |
|  | Diogenidae | Gobiidae | Alpheidae | Diogenidae | Ophiuroidea | Palaemonidae |  |
|  | Ophiuroidea | Diogenidae | Gobiidae | Galatheidae | Diogenidae | Tetraliidae |  |
|  | Gobiidae | Limidae | Muricidae | Potamididae | Ophiomyxidae | Alpheidae |  |
|  | Muricidae | Muricidae | Tetraliidae | Ophiactidae | Ophiactidae | Fasciolariidae |  |
|  | Pectinidae | Xanthidae | Trapeziidae | Alpheidae | Palaemonidae | Gobiidae | **NGK N** |
| **SDO** | 1.64, **0.005** | 1.66, **0.006** | 0.94, 0.546 | 1.09, 0.321 | 1.65, **0.002** | 1.16, 0.222 | 1.55, **0.011** |
|  | *60.2%* | *54.4%* | *55.0%* | *58.6%* | *58.1%* | *50.2%* | *56.2%* |
|  | Gobiidae | Palaemonidae | Gobiidae | Palaemonidae | Diogenidae | Gobiidae | Gobiidae |
|  | Palaemonidae | Gobiidae | Palaemonidae | Gobiidae | Gobiidae | Palaemonidae | Muricidae |
|  | Ophiuroidea | Limidae | Trapeziidae | Diogenidae | Muricidae | Diogenidae | Scorpaenidae |
|  | Ophiactidae | Xanthidae | Tetraliidae | Potamididae | Ophiuroidea | Ophiomyxidae | Diodenidae |
|  | Muricidae | Alpheidae | Ophiomyxidae | Galatheidae | Palaemonidae | Fasciolariidae | Alpheidae |
|  | Diogenidae | Diogenidae | Turbinidae | Alpheidae | Ophiactidae | Scorpaenidae | Ophiomyxidae |

**Table S6:** Results of SIMPER analysis of motile cryptofauna (by Family) that contributed most strongly to dissimilarities in biomass between locations and microhabitats. Families are listed in order of their contribution (top six) to dissimilarities. Grey cells show t- and p-values from Pairwise tests (PERMANOVA) with significant values in bold (df = 13–16, unique perms = 997–999); categories to the left of the cell were greater in the factor labelled by row, while categories to the right were greater in the factor labelled by column.

|  | **SEW 1** |  |  | ***Acropora*** | ***Pocillopora*** | **Dead coral** |  |
| --- | --- | --- | --- | --- | --- | --- | --- |
| **SEW 2** | 1.11, 0.265 |  |  | 5.49, **0.001** | 6.53, **0.001** | 2.79, **0.001** | **Rubble** |
|  | *57.9%* |  |  | *90.2%* | *89.2%* | *72.5%* |  |
|  | Muricidae |  |  | Tetraliidae | Trapeziidae | Diogenidae |  |
|  | Gobiidae |  |  | Galatheidae | Muricidae | Ophidiasteridae |  |
|  | Limidae |  |  | Potamididae | Galatheidae | Gobiidae |  |
|  | Palaemonidae |  |  | Haliotidae | Alpheidae | Galatheidae |  |
|  | Mytilidae |  |  | Fasciolariidae | Diogenidae | Haliotidae |  |
|  | Ophidiasteridae | **SEW 2** |  | Palaemonidae | Gobiidae | Potamididae |  |
| **SEW 3** | 1.28, 0.095 | 1.50, **0.011** |  |  | 6.54, **0.001** | 5.06, **0.001** | ***Acropora*** |
|  | *60.7%* | *61.3%* |  |  | *87.3%* | *91.8%* |  |
|  | Muricidae | Gobiidae |  |  | Trapeziidae | Tetraliidae |  |
|  | Diogenidae | Diogenidae |  |  | Tetraliidae | Diogenidae |  |
|  | Gobiidae | Trapeziidae |  |  | Alpheidae | Xanthidae |  |
|  | Trapeziidae | Tetraliidae |  |  | Muricidae | Galatheidae |  |
|  | Palaemonidae | Limidae |  |  | Diogenidae | Gobiidae |  |
|  | Mytilidae | Palaemonidae | **SEW 3** |  | Gobiidae | Muricidae |  |
| **SEW 4** | 1.12, 0.259 | 1.34, **0.032** | 0.84, 0.717 |  |  | 5.39, **0.001** | ***Pocillopora*** |
|  | *59.6%* | *57.4%* | *58.7%* |  |  | *83.9%* |  |
|  | Muricidae | Diogenidae | Diogenidae |  |  | Trapeziidae |  |
|  | Diogenidae | Muricidae | Trapeziidae |  |  | Muricidae |  |
|  | Palaemonidae | Gobiidae | Alpheidae |  |  | Alpheidae |  |
|  | Gobiidae | Limidae | Gobiidae |  |  | Xanthidae |  |
|  | Pectinidae | Palaemonidae | Tetraliidae |  |  | Diogenidae |  |
|  | Trapeziidae | Trapeziidae | Palaemonidae | **SEW 4** |  | Galatheidae |  |
| **NGK S** | 1.37, 0.051 | 1.60, **0.007** | 1.18, 0.160 | 0.97, 0.491 |  |  |  |
|  | *64.0%* | *63.9%* | *62.1%* | *61.0%* |  |  |  |
|  | Diogenidae | Diogenidae | Diogenidae | Diogenidae |  |  |  |
|  | Muricidae | Muricidae | Ophiomyxidae | Muricidae |  |  |  |
|  | Ophidiasteridae | Ophidiasteridae | Muricidae | Ophidiasteridae |  |  |  |
|  | Alpheidae | Conidae | Alpheidae | Xanthidae |  |  |  |
|  | Palaemonidae | Columbellidae | Ophidiasteridae | Alpheidae |  |  |  |
|  | Ophiomyxidae | Ophiomyxidae | Columbellidae | Ophiomyxidae | **NGK S** |  |  |
| **NGK M** | 1.00, **0.002** | 2.35, **0.001** | 1.14, 0.229 | 1.40, **0.040** | 1.42, **0.041** |  |  |
|  | *62.5%* | *59.6%* | *54.5%* | *58.6%* | *59.1%* |  |  |
|  | Diogenidae | Diogenidae | Diogenidae | Diogenidae | Diogenidae |  |  |
|  | Muricidae | Gobiidae | Tetraliidae | Tetraliidae | Muricidae |  |  |
|  | Gobiidae | Muricidae | Gobiidae | Trapeziidae | Ophidiasteridae |  |  |
|  | Palaemonidae | Limidae | Trapeziidae | Trochidae | Ophiocomidae |  |  |
|  | Mytilidae | Palaemonidae | Ophiocomidae | Palaemonidae | Ophiomyxidae |  |  |
|  | Fasciolariidae | Tetraliidae | Muricidae | Gobiidae | Trochidae | **NGK M** |  |
| **NGK N** | 1.81, **0.003** | 2.06, **0.002** | 1.31, 0.080 | 1.17, 0.174 | 1.08, 0.337 | 1.35, 0.088 |  |
|  | *63.7%* | *60.0%* | *50.2%* | *57.2%* | *58.5%* | *51.0%* |  |
|  | Diogenidae | Diogenidae | Diogenidae | Diogenidae | Diogenidae | Diogenidae |  |
|  | Muricidae | Muricidae | Muricidae | Muricidae | Muricidae | Muricidae |  |
|  | Palaemonidae | Gobiidae | Trapeziidae | Trapeziidae | Ophidiasteridae | Tetraliidae |  |
|  | Gobiidae | Limidae | Gobiidae | Alpheidae | Ophiomyxidae | Palaemonidae |  |
|  | Mytilidae | Palaemonidae | Tetraliidae | Palaemonidae | Alpheidae | Fasciolariidae |  |
|  | Ophidiasteridae | Conidae | Alpheidae | Ophidiasteridae | Ophiuroidea | Gobiidae | **NGK N** |
| **SDO** | 1.25, 0.140 | 1.62, **0.004** | 0.86, 0.729 | 0.97, 0.522 | 1.35, **0.049** | 1.39, 0.069 | 1.29, 0.098 |
|  | *62.3%* | *60.2%* | *59.1%* | *61.9%* | *65.6%* | *54.9%* | *58.7%* |
|  | Muricidae | Diogenidae | Gobiidae | Diogenidae | Diogenidae | Diogenidae | Diogenidae |
|  | Diogenidae | Palaemonidae | Trapeziidae | Alpheidae | Muricidae | Gobiidae | Muricidae |
|  | Gobiidae | Muricidae | Diogenidae | Palaemonidae | Alpheidae | Muricidae | Gobiidae |
|  | Palaemonidae | Limidae | Tetraliidae | Gobiidae | Potamididae | Ophiomyxidae | Scorpaenidae |
|  | Scorpaenidae | Gobiidae | Muricidae | Trapeziidae | Ophidiasteridae | Palaemonidae | Ophiomyxidae |
|  | Potamididae | Scorpaenidae | Ophiomyxidae | Potamididae | Turbinidae | Scorpaenidae | Alpheidae |

**Table S7:** Results of SIMPER analysis of motile cryptofauna (by functional group) that contributed most strongly to dissimilarities in density between locations and microhabitats. Groups are listed in order of their contribution (top six) to dissimilarities. Grey cells show t- and p-values from Pairwise tests (PERMANOVA) with significant values in bold (df = 13–16, unique perms = 997–999); categories to the left of the cell were greater in the factor labelled by row, while categories to the right were greater in the factor labelled by column.

|  | **SEW 1** |  |  | ***Acropora*** | ***Pocillopora*** | **Dead coral** |  |
| --- | --- | --- | --- | --- | --- | --- | --- |
| **SEW 2** | 1.42, 0.111 |  |  | 10.17, **0.001** | 9.09, **0.001** | 3.64, **0.001** | **Rubble** |
|  | *33.3%* |  |  | *74.4%* | *58.9%* | *30.7%* |  |
|  | NCE |  |  | OG | OG | PG |  |
|  | FISH |  |  | PC | PG | FISH |  |
|  | BIV |  |  | SW | SW | SBI |  |
|  | SW |  |  | SG | SPC | SC |  |
|  | SG |  |  | PG | PC | SG |  |
|  | SC | **SEW 2** |  | SC | FISH | OG |  |
| **SEW 3** | 1.76, **0.025** | 1.43, 0.111 |  |  | 4.16, **0.001** | 7.76, **0.001** | ***Acropora*** |
|  | *37.5%* | *36.8%* |  |  | *51.6%* | *73.2%* |  |
|  | BIV | NCE |  |  | SC | PC |  |
|  | NCE | FISH |  |  | SG | SPC |  |
|  | SC | BIV |  |  | FISH | SW |  |
|  | PC | PC |  |  | PC | SG |  |
|  | FISH | SC |  |  | NCE | OG |  |
|  | SG | HC | **SEW 3** |  | HC | SC |  |
| **SEW 4** | 1.16, 0.276 | 1.19, 0.232 | 0.74, 0.731 |  |  | 6.06, **0.001** | ***Pocillopora*** |
|  | *33.2%* | *34.2%* | *31.9%* |  |  | *53.7%* |  |
|  | BIV | FISH | PC |  |  | SPC |  |
|  | SG | SG | SG |  |  | PC |  |
|  | PC | BIV | NCE |  |  | OG |  |
|  | NCE | NCE | SC |  |  | SW |  |
|  | SC | SC | FISH |  |  | PG |  |
|  | HC | PC | HC | **SEW 4** |  | BIV |  |
| **NGK S** | 1.82, **0.043** | 1.37, 0.173 | 0.69, 0.737 | 0.31, 0.914 |  |  |  |
|  | *32.8%* | *27.4%* | *26.5%* | *24.6%* |  |  |  |
|  | HC | HC | NCE | FISH |  |  |  |
|  | BIV | NCE | FISH | SG |  |  |  |
|  | FISH | BIV | SG | HC |  |  |  |
|  | SG | FISH | HC | PC |  |  |  |
|  | SW | PG | SC | NCE |  |  |  |
|  | NCE | SG | PC | SC | **NGK S** |  |  |
| **NGK M** | 2.12, **0.008** | 1.92, **0.022** | 0.86, 0.571 | 0.76, 0.604 | 0.71, 0.606 |  |  |
|  | *36.2%* | *35.6%* | *29.5%* | *29.2%* | *23.0%* |  |  |
|  | HC | HC | PC | HC | HC |  |  |
|  | BIV | FISH | FISH | PC | FISH |  |  |
|  | PC | BIV | SG | SG | PC |  |  |
|  | NCE | PC | NCE | OG | SG |  |  |
|  | SG | SG | HC | SPC | PG |  |  |
|  | SC | SPC | SPC | FISH | OG | **NGK M** |  |
| **NGK N** | 2.30, **0.006** | 1.63, 0.069 | 1.43, 0.115 | 1.59, 0.066 | 1.27, 0.221 | 1.26, 0.244 |  |
|  | *38.6%* | *37.6%* | *33.0%* | *33.2%* | *26.7%* | *28.9%* |  |
|  | BIV | FISH | NCE | PC | NCE | HC |  |
|  | NCE | PC | SG | SG | FISH | SPC |  |
|  | HC | BIV | FISH | HC | SG | NCE |  |
|  | SG | HC | SC | NCE | HC | PC |  |
|  | PC | SG | HC | OG | PG | SG |  |
|  | SC | NCE | PG | SC | PC | FISH | **NGK N** |
| **SDO** | 1.76, 0.054 | 1.42, 0.136 | 0.38, 0.871 | 0.72, 0.691 | 0.56, 0.789 | 0.94, 0.486 | 1.40, 0.140 |
|  | *63.9%* | *36.5%* | *30.5%* | *34.1%* | *26.3%* | *27.2%* | *32.2%* |
|  | BIV | PC | NCE | PC | FISH | FISH | FISH |
|  | NCE | FISH | FISH | SG | HC | NCE | NCE |
|  | FISH | BIV | PC | NCE | NCE | HC | SG |
|  | PC | NCE | PG | HC | SG | PC | HC |
|  | SW | HC | HC | FISH | SW | SG | SC |
|  | SG | PG | SC | OG | OG | SPC | SPC |

**Table S8:** Results of SIMPER analysis of motile cryptofauna (by functional group) that contributed most strongly to dissimilarities in biomass between locations and microhabitats. Groups are listed in order of their contribution (top six) to dissimilarities. Grey cells show t- and p-values from Pairwise tests (PERMANOVA) with significant values in bold (df = 13–16, unique perms = 997–999); categories to the left of the cell were greater in the factor labelled by row, while categories to the right were greater in the factor labelled by column.

|  | **SEW 1** |  |  | ***Acropora*** | ***Pocillopora*** | **Dead coral** |  |
| --- | --- | --- | --- | --- | --- | --- | --- |
| **SEW 2** | 0.77, 0.679 |  |  | 8.67, **0.001** | 7.22, **0.001** | 3.04, **0.001** | **Rubble** |
|  | *42.9%* |  |  | *80.7%* | *66.7%* | *47.5%* |  |
|  | SG |  |  | SPC | SPC | PG |  |
|  | FISH |  |  | PG | PC | HC |  |
|  | BIV |  |  | PC | PG | SG |  |
|  | NCE |  |  | SG | OG | OG |  |
|  | PC |  |  | OG | SG | FISH |  |
|  | OG | **SEW 2** |  | HC | HC | NCE |  |
| **SEW 3** | 1.70, **0.027** | 1.60, **0.043** |  |  | 4.78, **0.001** | 7.44, **0.001** | ***Acropora*** |
|  | *44.3%* | *41.5%* |  |  | *56.5%* | *82.8%* |  |
|  | BIV | HC |  |  | SC | SPC |  |
|  | HC | FISH |  |  | SG | PC |  |
|  | SG | BIV |  |  | HC | HC |  |
|  | NCE | NCE |  |  | FISH | SG |  |
|  | FISH | PC |  |  | SPC | FISH |  |
|  | PC | SG | **SEW 3** |  | NCE | NCE |  |
| **SEW 4** | 1.26, 0.171 | 0.73, 0.726 | 0.70, 0.819 |  |  | 5.92, **0.001** | ***Pocillopora*** |
|  | *44.6%* | *42.6%* | *38.3%* |  |  | *67.0%* |  |
|  | SG | SG | HC |  |  | SPC |  |
|  | BIV | HC | SC |  |  | SG |  |
|  | HC | NCE | SG |  |  | PC |  |
|  | NCE | BIV | FISH |  |  | SC |  |
|  | PC | PC | PC |  |  | HC |  |
|  | FISH | FISH | SPC | **SEW 4** |  | NCE |  |
| **NGK S** | 1.76, **0.025** | 2.01, **0.007** | 1.70, 0.055 | 1.22, 0.218 |  |  |  |
|  | *45.8%* | *43.6%* | *39.6%* | *40.5%* |  |  |  |
|  | HC | SG | HC | HC |  |  |  |
|  | SG | NCE | SG | NCE |  |  |  |
|  | NCE | HC | NCE | SG |  |  |  |
|  | SC | OG | SC | PG |  |  |  |
|  | FISH | BIV | FISH | SC |  |  |  |
|  | BIV | FISH | PG | FISH | **NGK S** |  |  |
| **NGK M** | 2.24, **0.007** | 2.37, **0.005** | 1.01, 0.401 | 1.23, 0.215 | 1.85, **0.036** |  |  |
|  | *47.8%* | *45.0%* | *36.2%* | *42.1%* | *40.1%* |  |  |
|  | HC | HC | HC | HC | HC |  |  |
|  | BIV | SG | NCE | NCE | NCE |  |  |
|  | SG | FISH | SG | SG | SG |  |  |
|  | NCE | BIV | FISH | PG | PG |  |  |
|  | PC | PC | PC | PC | FISH |  |  |
|  | PG | OG | PG | OG | SC | **NGK M** |  |
| **NGK N** | 1.89, **0.017** | 1.75, **0.038** | 1.37, 0.136 | 1.30, 0. 719 | 1.66, 0.072 | 1.45, 0.119 |  |
|  | *49.4%* | *45.6%* | *40.6%* | *41.7%* | *40.9%* | *38.2%* |  |
|  | HC | HC | SG | HC | HC | HC |  |
|  | SG | SG | HC | SG | NCE | SG |  |
|  | BIV | BIV | FISH | NCE | SG | NCE |  |
|  | NCE | FISH | NCE | PC | SC | SPC |  |
|  | FISH | PC | SPC | PG | FISH | PG |  |
|  | PC | OG | SC | SC | BIV | PC | **NGK N** |
| **SDO** | 1.34, 0.131 | 1.14, 0.298 | 0.52, 0.884 | 0.98, 0.414 | 1.89, **0.014** | 1.33, 0.164 | 1.20, 0.256 |
|  | *47.5%* | *44.1%* | *36.9%* | *42.5%* | *45.0%* | *38.2%* | *42.0%* |
|  | SG | FISH | NCE | HC | NCE | HC | HC |
|  | BIV | HC | FISH | NCE | HC | SG | SG |
|  | FISH | SG | HC | FISH | SG | NCE | FISH |
|  | HC | BIV | SG | SG | OG | FISH | NCE |
|  | NCE | PC | OG | OG | PG | PG | PG |
|  | PC | OG | SC | PC | FISH | OG | OG |
